# Comprehensive characterization of the cellular and molecular consequences of LCN2 loss in MDA-MB-231 triple-negative breast cancer cells

**DOI:** 10.3389/fcell.2026.1909534

**Published:** 2026-10-21

**Authors:** Diandra T. Keller, Stefanie Kankel, Thomas Liehr, Katharina S. Hardt, Eva M. Buhl, Ralf Weiskirchen, Sarah K. Schröder-Lange

**Affiliations:** 1 Institute for Molecular Pathobiochemistry, Experimental Gene Therapy and Clinical Chemistry (IFMPEGKC), RWTH University Hospital Aachen, Aachen, Germany; 2 Jena University Hospital, Institute of Human Genetics, Friedrich-Schiller University, Jena, Germany; 3 Electron Microscopy Facility, Institute of Pathology, RWTH University Hospital Aachen, Aachen, Germany

**Keywords:** CRISPR/Cas9, LCN2, MDA-MB-231, metastasis, TNBC, transcriptomic analysis

## Abstract

**Introduction:**

Triple-negative breast cancer (TNBC) is an aggressive breast cancer subtype characterized by high metastatic potential and limited targeted therapeutic options. Lipocalin-2 (LCN2) has been implicated in tumor progression and metastatic dissemination, but its functional role in TNBC remains incompletely understood. This study investigated the role of LCN2 in the human TNBC cell line MDA-MB-231.

**Methods:**

Stable LCN2-deficient (LCN2KO) cells were generated using CRISPR/Cas9 genome editing, followed by isolation of single-cell-derived clones. RNA sequencing was performed to characterize transcriptomic changes, with selected findings validated by reverse transcription quantitative PCR (RT-qPCR). Western blotting was used to assess protein-level changes. Transwell migration and invasion assays and adhesion assays were performed to evaluate functional effects.

**Results:**

LCN2KO cells largely retained the mesenchymal phenotype of parental cells. RNA sequencing revealed changes associated with inflammatory signaling, iron homeostasis, oxidative stress, ferroptosis-related processes, and cellular metabolism. Functional assays demonstrated reduced migration, invasion, and adhesion, accompanied by altered adhesion to extracellular matrix components.

**Discussion:**

LCN2 deficiency is associated with transcriptional and functional changes in MDA-MB-231 cells, particularly affecting migratory and invasive behavior and cell–matrix interactions, while largely preserving the mesenchymal phenotype. These findings provide further insight into the role of LCN2 in cellular processes relevant to TNBC progression and metastatic behavior.

## 1 Introduction

Breast cancer is the most common cancer among women worldwide and a major cause of cancer mortality (Sung et al., 2026). It can be subdivided into four molecular subtypes based on the expression of hormone receptors (estrogen, progesterone, human epidermal growth factor receptor 2 (HER2)): luminal A, luminal B, HER2^+^ and triple-negative. Triple-negative breast cancer (TNBC) is characterized by a lack of expression of estrogen and progesterone receptors, as well as HER2, limiting its treatment options (Schwentner et al., 2012). Although only 10%–17% of all breast cancer cases are classified as TNBC, this subtype displays disproportionately aggressive and clinically challenging behavior. This is accompanied by early metastasis, leading to a poor prognosis (Sheppard et al., 2017).

Lipocalin-2 (LCN2) is a 25-kDa secreted protein, also known as neutrophil gelatinase-associated lipocalin (NGAL). It is a member of the lipocalin superfamily and shares a highly conserved eight-stranded antiparallel β-barrel structure (Flower, 1996). LCN2 is a highly versatile protein that exerts several physiological and pathological functions. Physiologically, it acts as a transporter protein for hydrophobic molecules, including fatty acids, steroids, hormones and retinoids (Flower, 1996). Furthermore, it plays a role in the innate immune system by binding enterochelin secreted by bacteria, thereby sequestering iron (Flo et al., 2004). In pathological context, cancer cells can exploit its iron-binding capacity, which is linked to tumor progression, metastasis, and recurrence (Chi et al., 2020). Additionally, human LCN2 interacts with matrix metalloproteinase-9 (MMP-9), forming an LCN2–MMP-9 complex through a disulfide linkage involving LCN2 and the propeptide region of MMP-9 (Yan et al., 2001; Wu et al., 2008; Schröder et al., 2023a). Formation of this complex protects MMP-9 from autodegradation and thereby preserves its extracellular proteolytic activity (Kubben et al., 2007). As MMP-9 contributes to extracellular matrix degradation and remodeling, the LCN2–MMP-9 axis has been implicated in tumor cell migration and invasion. More recent studies have provided evidence for both intracellular and extracellular LCN2–MMP-9 interactions and have linked this association to ECM degradation, cytoskeletal remodeling, and invasive behavior (Zhao et al., 2024; Xia et al., 2023). Consequently, the LCN2–MMP-9 interaction represents a potential mechanism by which LCN2 may influence cell–matrix interactions and tumor cell invasion.

Although LCN2 is predominantly characterized as a secreted protein, accumulating evidence indicates that it also undergoes intracellular trafficking and localizes to distinct subcellular compartments. Following synthesis in the endoplasmic reticulum (ER), LCN2 is transported through the secretory pathway. Furthermore, it has been detected within endosomal compartments, including Rab7- and Rab9-positive vesicles, where it has been implicated in the regulation of receptor trafficking (Yammine et al., 2019). Beyond its functions in extracellular signaling and matrix remodeling, accumulating evidence suggests that LCN2 may also influence the organization of the actin cytoskeleton and cell–matrix interactions (Choudhary et al., 2023; Choudhary et al., 2025). LCN2 has been reported to promote actin polymerization and to alter F-actin organization through signaling pathways involving actin glutathionylation, ERK, cofilin, and ERM proteins (Choudhary et al., 2023). More recently, LCN2 has also been associated with focal adhesion formation through signaling involving c-Src and paxillin (Choudhary et al., 2025). These observations suggest that LCN2 may regulate cellular migration and adhesion through indirect effects on actin dynamics and focal adhesion-associated signaling. However, a direct physical interaction between LCN2 and actin has not been established.

Aberrant LCN2 expression is observed in neoplastic tissues, including breast cancer (Yang et al., 2009). Depending on the tissue of origin, it can act either as a tumor-promoting factor (e.g., in non-small cell lung cancer) (Zhang et al., 2025; Nuntagowat et al., 2010) or as a tumor-suppressing factor (e.g., hepatocellular carcinoma and pancreatic cancer) (Wani et al., 2014; Moniaux et al., 2008). Elevated LCN2 expression is associated with advanced disease stages and metastatic spread, highlighting its relevance to TNBC aggressiveness (Yang et al., 2009). Mechanistically, LCN2 upregulation in TNBC has been reported to be driven by oncogenic transcriptional programs, such as the Wilms tumor 1-associated protein/nuclear protein 1 (WTAP/NUPR1) axis (Tan et al., 2024), and reinforced by epigenetic alterations, including loss of repression mediated by hypermethylated in cancer 1 (HIC1) (Cheng et al., 2014; Parrella et al., 2005) and promoter hypomethylation (Meka et al., 2015). These regulatory events converge to sustain high LCN2 expression, which has been linked to increased metastatic potential and resistance to ferroptosis in TNBC cells. In addition, Yang and colleagues reported that LCN2 expression is associated with increased vascular endothelial growth factor (VEGF) expression (Yang et al., 2013), thereby promoting neovascularization and vascular permeability and contributing to metastatic dissemination. Knockout of LCN2 in cell lines, including MDA-MB-231 has been shown to diminish migration, alter ferroptosis sensitivity, and enhance chemotherapeutic vulnerability, underscoring its functional importance in cancer cell behavior (Valashedi et al., 2022). Complementary genetic knockout models in mice have revealed reduced tumor burden, delayed onset, and attenuated metastatic spread, highlighting the functional relevance of LCN2 *in vivo* (Leng et al., 2009). Despite structural differences between murine and human LCN2 (Holmes et al., 2005), these models have become valuable tools for dissecting the context-dependent roles of LCN2 and evaluating its potential as a prognostic biomarker and therapeutic target in TNBC research. Nevertheless, the precise molecular mechanisms by which LCN2 promotes progression and metastatic dissemination in TNBC remain incompletely defined. Given the context-dependent and sometimes divergent functions of LCN2 across tumor entities, a TNBC-specific dissection of its downstream signaling networks is essential to clarify its mechanistic contribution and to evaluate its potential as a biomarker or therapeutic target.

Using a stable CRISPR/Cas9-mediated LCN2 knockout model, we performed single-cell isolation and subsequently expanded and characterized individual single-cell-derived clones for downstream analyses. In this study, we characterized changes in cellular morphology and phenotype, transcriptomic profiles, and functional properties, including migration and adhesion. Particular attention was given to transcriptional changes associated with inflammatory signaling, iron homeostasis, oxidative stress, ferroptosis-related processes, and extracellular matrix interactions. By integrating transcriptomic and functional analyses, we aimed to define cellular processes associated with LCN2 deficiency and to further clarify its potential contribution to the migratory, invasive, and adhesive properties of MDA-MB-231 cells.

## 2 Materials and methods

### 2.1 Cell culture and light microscopic analysis

The TNBC cell lines MDA-MB-231 (RRID: CVCL_0062) and MDA-MB-468 (RRID: CVCL_0419) were kindly provided by Dr. Jochen Maurer (Department of Obstetrics and Gynecology, University Hospital Aachen, 52074 Aachen, Germany). The luminal-A breast cancer cell line MCF-7 (RRID: CVCL_0031) was kindly provided by Prof. Twan Lammers (Department of Nanomedicine and Theranostics, Institute for Experimental Molecular Imaging (ExMI), RWTH Aachen University Hospital, 52,074 Aachen, Germany). All cell lines were originally obtained from the American Type Culture Collection (ATCC, Manassas, VA, United States of America). Cell lines were cultured in Dulbecco’s Modified Eagle Medium (DMEM, high glucose, #D6171-500ML, Sigma-Aldrich, Merck, Taufkirchen, Germany) supplemented with 10% fetal bovine serum (FBS, #F7524, Sigma-Aldrich, Germany), 1× penicillin-streptomycin antibiotic solution (P0781-100ML, Sigma Life Science, Lonza, Cologne, Germany), 2 mM L-glutamine solution (#G7513, Sigma–Aldrich) and 1 mM sodium pyruvate solution (#S8636-100ML, Sigma-Aldrich). This medium is hereafter referred to as complete DMEM. All cell lines were routinely cultured in 10 cm dishes in a humidified incubator at 37 °C with 5% CO_2_ and passaged when they reached 80%–90% confluence using Accutase® solution (#A6964-100ML, Sigma-Aldrich). Cell lines were routinely tested for *mycoplasma* contamination (Venor®GeM OneStep, #11-8050, Minerva Biolabs®, Berlin, Germany). Only cells at passage numbers below 30 were used for experiments. Live-cell morphology was routinely observed by phase-contrast microscopy using a Leica DM IL LED microscope (Leica Camera, Wetzlar, Germany) with a Leica EC3 camera and the Leica Application Suite (LAS) software (version 3.4.0). Enlarged cell formation was assessed in bright-field images obtained from LCN2KO #15 (passage <10). Enlarged cells were operationally defined as adherent cells exhibiting a projected cell area at least twofold greater than the mean projected cell area of the corresponding parental MDA-MB-231 cells. This threshold was predefined before analysis to enable reproducible and unbiased classification, as no universally accepted morphological size criterion exists for the identification of enlarged tumor cells (Amend et al., 2019). Three representative fields per biological replicate were analyzed manually. The percentage of enlarged cells was calculated by dividing the number of enlarged cells by the total number of adherent cells in each field.

### 2.2 Generation of CRISPR/Cas9-mediated LCN2 knockout

A human LCN2 knockout kit (#KN207685, OriGene Technologies, Herford, Germany) was used to generate LCN2 knockout (LCN2KO) clones from MDA-MB-231 cells. A total of 500,000 cells were seeded in six-well cell culture plates. After 24 h, cells were co-transfected with a donor DNA plasmid (LoxP-EF1A-tGFP-P2A-Puro-LoxP) and two guide RNA (gRNA) vectors (gRNA1: ACA​GGG​CTA​GGC​CCA​GCC​AC, gRNA2: AGA​GGG​ACC​TTG​CTC​AGA​GG) using Lipofectamine™ 2000 (#11668019, Invitrogen, Thermo Fisher Scientific, Waltham, MA, United States of America) in serum-free Opti-MEM™ medium (#31985070, Thermo Fisher Scientific).

### 2.3 Transient transfection

For transient transfection, 500,000 cells were seeded in duplicate in six-well plates. Transfection was performed with FuGENE® 4K Transfection Reagent (#E5911, Promega, Madison, WI, United States of America) according to the manufacturer’s instructions. Cells were transfected at approximately 80% confluence with 2 µg plasmid DNA and 6 µL FuGENE® 4K Transfection Reagent diluted in serum-reduced Opti-MEM™ medium (#31985070, Thermo Fisher Scientific). The transfection mixture was added dropwise to the cells and incubated for at least 24 h at 37 °C and 5% CO_2_. Cells were transiently transfected with either pCMV-SPORT6-hLCN2 (LCN2 expression vector, #IMAGp998B166030Q, BioScience LifeSciences, Nottingham, United Kingdom) or pEGFP-N1 (GFP control vector, # 6085-1, Clontech, TaKaRa Bio Europe SAS, Göteborg, Sweden) (Schröder et al., 2022). Transfection efficiency was assessed by fluorescence microscopy based on GFP expression in pEGFP-N1-transfected cells.

### 2.4 Short tandem repeat profiling

Short tandem repeat (STR) profiling of the cell lines MDA-MB-231 and MDA-MB-468 used in this study was conducted using the cell line authentication service provided by IDEXX BioAnalytics in Kornwestheim, Germany. The CellCheck™ Human system chosen for this analysis includes 16 species-specific STR markers. Eight of these markers are based on guidelines from the ATCC, which recommends a minimum of eight core STR loci (CSF1PO, D13S317, D16S539, D5S818, D7S820, TH01, TPOX, and vWA), as well as the Amelogenin (AMEL) gene for sex determination (Almeida and Korch, 2004). Additionally, the CellCheck™ Human system includes seven additional markers (D18S51, D21S11, D3S1358, D8S1179, FGA, Penta D, and Penta E), which enhances the robustness of the analysis. Subsequent STR similarity searches were conducted using the Cellosaurus STR Similarity Search Tool, CLASTR 1.4.4, release 41.0 (Bairoch, 2018).

### 2.5 Combined karyotype and M-FISH analysis

Preparation of MDA-MB-231 and LCN2KO #15 metaphase chromosomes, karyotyping, and molecular cytogenetics were conducted according to established protocols by the Institute of Human Genetics, Friedrich-Schiller University, Jena (Liehr et al., 2024; Luis et al., 2025). Metaphase spreads were prepared from continuously growing cell cultures by transient mitotic arrest using a microtubule inhibitor (KaryoMAX colcemid solution, #15212012, Gibco, Thermo Fisher Scientific), hypotonic treatment, and fixation in methanol:acetic acid. Multicolor fluorescence *in situ* hybridization (M-FISH) was carried out with a probe mixture containing 24 whole chromosome painting probes specific to the 24 different human chromosomes (#D-0125–120-DI, XCyting, MetaSystems Probes, Altlussheim, Germany), following the manufacturer’s protocol to assign distinct colors to each homologous chromosome pair. A total of 20 metaphases were analyzed by M-FISH. Imaging was performed using a Zeiss Axioplan fluorescence microscope equipped with a CCD camera and ISIS software (ISIS, MetaSystems).

### 2.6 Electron microscopic cell analysis

For ultrastructure analysis, parental MDA-MB-231 and LCN2KO #15 cells were cultivated as described in Section 2.1. The cells were washed with 1× PBS, fixed with 2.5% glutaraldehyde in 0.1 M Sörensen’s phosphate buffer (#P4474, Sigma-Aldrich, Merck), and then post-fixed in 25 mM sucrose buffer containing a 1% osmium tetroxide solution (Carl Roth, Karlsruhe, Germany) following previously published protocols (Hardt et al., 2026; Schröder-Lange et al., 2025; Buitkamp et al., 2025). Briefly, the cells were dehydrated in graded ethanol series and embedded in epoxy resin. Ultrathin sections (70–90 nm, Reichert Ultracut S, Leica Camera) were prepared, mounted on copper grids (HR23 Maxtaform, Plano GmbH, Wetzlar, Germany), stained with 0.5% uranyl acetate and 1% lead citrate (both from Science Services, Munich, Germany) and examined using a Zeiss Leo 906 transmission electron microscope (Carl Zeiss AG, Oberkochen, Germany) at 60 kV. High-resolution images at magnifications ranging from 1,670× to 21,560× were acquired to assess organelle preservation and subcellular structures.

### 2.7 RNA extraction and real-time quantitative polymerase chain reaction (RT-qPCR)

A total of 300,000 cells were seeded in six-well cell culture plates and cultured for 72 h. Total RNA from all cell lines was purified using the PureLink™ RNA Mini Kit (#12183018A, Invitrogen, Thermo Fisher Scientific) according to the manufacturer’s protocol, including DNase digestion using the PureLink™ DNase Set (#12185-010, Thermo Fisher Scientific). The protocols for complementary DNA (cDNA) synthesis and real-time quantitative PCR (RT-qPCR) have been described previously (Hardt et al., 2026; Kessel et al., 2023). A total of 1 µg of RNA was reverse-transcribed into cDNA using SuperScript™ II Reverse Transcriptase (#18064022, Invitrogen, Thermo Fisher Scientific) and random hexadeoxynucleotides (#C1181, Promega) as primers. For RT-qPCR, 25 ng of cDNA and 20 pmol of each primer were mixed with 20 µL SYBR-GreenER™ qPCR Super Mix Universal (#11760500, Thermo Fisher Scientific). Specific primers are listed in Supplementary Table S1. The qPCR cycling conditions consisted of an initial denaturation step at 95 °C for 10 min, followed by 40 amplification cycles comprising denaturation at 95 °C for 15 s and annealing/extension at 60 °C for 1 min. Each sample was measured in duplicate. Relative mRNA expression normalized to *GAPDH* was calculated using the 2^−ΔΔCT^ method (Livak and Schmittgen, 2001).

### 2.8 Next-generation sequencing and data analysis

Next-generation sequencing (NGS) analysis of parental MDA-MB-231 cells and LCN2KO #15 cells was conducted according to an established protocol (Liehr et al., 2024) with minor deviations. RNA from parental and KO cells was collected from three independent biological experiments (n = 3) as described in Section 2.7. RNA concentration, purity, and integrity were evaluated using UV spectrophotometry using the Agilent 4200 TapeStation system (Agilent Technologies Inc., Waldbronn, Germany). Ribosomal RNA was depleted, and mRNA was converted into sequencing libraries using the NEBNext Multiplex Oligos for Illumina Index Primer Set 1 kit (#E7335, New England Biolabs, Frankfurt am Main, Germany). 3′mRNA sequencing was carried out on an Illumina platform using prefilled MiSeq Reagent Kit v2 cartridges (300 cycles, Illumina Inc., San Diego, CA, United States of America). Sequencing output files were converted into FASTQ format for further analysis. Library preparation and sequencing were performed at the IZKF Genomic Facility, University Hospital Aachen. Data were processed using nf-core/rnaseq v3.22.2 from the nf-core collection of workflows (Ewels et al., 2020), utilizing reproducible software environments from the Bioconda (Grüning et al., 2018) and Biocontainers (da Veiga Leprevost et al., 2017) projects. Differential gene expression analysis was performed using the DESeq2 package in R (Love et al., 2014). Genes were considered differentially expressed when the adjusted *p*-value was <0.05 and the absolute log_2_ fold change (log_2_fc) was ≥1.

To categorize differentially expressed genes according to biological processes, Gene Ontology (GO) Biological Process (GO-BP) enrichment analysis was performed using the clusterProfiler package in R (Wu et al., 2021). Overrepresentation analysis (ORA) was used to identify significantly overrepresented GO-BP terms among the predefined list of differentially expressed genes. Statistical significance was assessed using a hypergeometric test with adjustment for multiple testing. The biological-process categories presented in Supplementary Table S5 were defined based on the significantly enriched GO-BP terms and the genes associated with these terms.

### 2.9 Western blot analysis

Proteins were extracted from cell lines according to established protocols (Hardt et al., 2026). Equal amounts of protein (30 µg) were denatured at 80 °C for 10 min under reducing conditions in 4× NuPAGE™ LDS buffer (#NP0007, Thermo Fisher Scientific). The proteins were separated using sodium dodecyl sulfate-polyacrylamide gel electrophoresis (SDS-PAGE) on 4%–12% Bis-Tris gradient gels (#NP0335BOX or #WG1402BOX, Thermo Fisher Scientific) and 2-(N-morpholino)ethanesulfonic acid (MES) running buffer. The proteins were transferred onto a nitrocellulose membrane (#GE10600002, 0.45 μm, Merck, Darmstadt, Germany) in 1× NuPAGE™ transfer buffer (#NP0006, Thermo Fisher Scientific). Equal protein loading and successful transfer were confirmed by Ponceau S staining. The membranes were blocked with 5% (w/v) non-fat milk powder in Tris-buffered saline with Tween® 20. Primary antibodies were incubated overnight at 4 °C with gentle agitation. Chemiluminescence was visualized using secondary antibodies coupled to horseradish peroxidase and SuperSignal™ West Dura Extended Duration Substrate (#34076, Thermo Fisher Scientific). The antibodies used are listed in Supplementary Table S2.

### 2.10 Immunofluorescence and F-Actin staining

Staining of F-actin fibers was performed according to established protocols (Schröder-Lange et al., 2025; Schröder et al., 2023b). A total of 30,000 cells per well were plated onto four-chamber polystyrene vessel tissue culture–treated glass slides (#354114, Falcon®, Corning Life Science, Tewksbury, MA, United States of America). Once cells reached approximately 80% confluence, they were rinsed with 1× PBS and fixed using 4% paraformaldehyde for 20 min. Permeabilization was then performed with 0.2% Triton X-100 in PBS for 4 min on ice. To reduce non-specific antibody binding, samples were incubated in 3% bovine serum albumin (BSA) in PBS for 1 h at room temperature. F-actin structures were visualized by staining with rhodamine-conjugated phalloidin (#R415, Invitrogen™, Thermo Fisher Scientific) for 20 min in the dark. Cell nuclei were counterstained with 4′,6-diamidino-2-phenylindole (DAPI, #D1306, Thermo Fisher Scientific) for 30 min. Finally, slides were mounted using VECTASHIELD® Antifade Mounting Medium (#H-1000-10, Vector Laboratories, Burlingame, CA, United States of America) and stored protected from light at 4 °C until imaging. Fluorescence imaging was performed using a Nikon Eclipse E80i microscope equipped with NIS-Elements Vis software (Version 3.22.01, Nikon Instruments, Düsseldorf, Germany).

### 2.11 Proliferation assay

Proliferation was measured using an ATP-based luminescence assay. The CellTiter-Glo®3D Cell Viability Assay (#G9683, Promega) was used according to the manufacturer’s protocol. Briefly, 2,000 cells were cultured in triplicate in 100 µL complete medium in opaque 96-well plates (#353948, BD Falcon™, BD Bioscience, Franklin Lakes, NJ, United States of America) for 24 and 48 h at 37 °C and 5% CO_2_. The CellTiter-Glo®3D reagent and the plate were equilibrated to room temperature for 30 min. A total of 100 µL CellTiter-Glo®3D reagent was added to each well and mixed for 5 min. The plate was incubated for 25 min at room temperature, after which, luminescence was measured using a Victor X3 Multiplate Reader (Perkin Elmer, MA, United States of America). Proliferation was assessed in three independent experiments.

### 2.12 Transwell migration assay

Migration of cells was studied using a Transwell migration assay (Justus et al., 2023). A total of 500,000 cells were resuspended in migration medium (DMEM without FBS) and transferred to MilliCell® Cell Culture Inserts with 8.0 µm PET membranes (#PCEP06H48, Merck), in triplicate. Cells were incubated at 37 °C for 10 min. Complete DMEM was added to the well directly below the insert and cells were further incubated at 37 °C for 6 h. A cotton-tipped applicator was used to gently remove any non-migrated cells from the apical side of the Transwell insert. The Transwell insert was allowed to dry and the remaining cells on the basal side were stained with 0.2% (w/v) crystal violet (#C0775, Sigma-Aldrich) and subsequently washed twice with 1× PBS. Ten representative fields of view from each insert were imaged using a Leica DM IL LED microscope with a Leica EC3 camera. Migrated cells were counted using ImageJ (National Institutes of Health, Bethesda, MD, United States of America) (Schneider et al., 2012). Migration was assessed in three independent experiments (n = 3).

### 2.13 Transwell invasion assay

The invasive capacity of parental MDA-MB-231 cells and LCN2KO clones #7 and #15 was investigated using a Transwell invasion assay (Justus et al., 2023). Prior to cell seeding, MilliCell® Cell Culture Inserts with 8.0 µm PET membranes (#PCEP06H48, Merck) were coated with 1 mg/mL Geltrex™ Flex LDEV-Free Reduced Growth Factor Basement Membrane Matrix (#A4000046703, Gibco, Thermo Fisher Scientific) and incubated for 1 h at 37 °C. A total of 300,000 cells were resuspended in migration medium and transferred to the Transwell inserts. Cells were incubated at 37 °C for 10 min, after which complete DMEM was added to the lower well directly below the insert, and the cells were incubated for 12 h at 37 °C. Invaded cells were stained and analyzed as described in Section 2.12. Invasion was assessed in three independent biological experiments (n = 3).

### 2.14 Cell adhesion assay

The cell adhesion assay was performed in 48-well plates (#150687, Thermo Fisher Scientific). The wells were coated with 0.1% Geltrex (#A14132-02, Invitrogen, Thermo Fisher Scientific), 50 μg/mL rat tail collagen type I (#A1048301, Gibco, Thermo Fisher Scientific) or 0.1% gelatin (#0621.1, Carl Roth). The coatings were incubated for 1 h at 37 °C. A total of 50,000 cells of each cell line were added to the indicated wells in triplicate and incubated for 1 h. Subsequently, the wells were washed twice with 1× PBS, and adherent cells were fixed with ice-cold 100% methanol for 10 min. A 0.2% crystal violet (w/v) solution was added and incubated for 10 min. Then, the wells were washed to remove excess dye. Crystal violet in attached cells was dissolved with 100% methanol, and absorbance was measured at 590 nm using the Victor X3 Multiplate Reader (Perkin Elmer, MA, United States of America). Adhesion was assessed in three independent experiments (n = 3).

### 2.15 Statistics

Statistical analysis was conducted using GraphPad Prism 8 (GraphPad Software, Boston, MA, United States of America). The data were assessed for normality using the Shapiro–Wilk test. For datasets meeting the assumption of normal distribution, a one-way ANOVA followed by Dunnett’s multiple comparisons test or an unpaired *t*-test was applied. In cases where normality was not satisfied, a non-parametric Kruskal-Wallis test followed by Dunn’s multiple comparisons test or a non-parametric Mann-Whitney U test was conducted. Data are presented as the mean ± standard deviation (SD). Statistical significance was indicated with asterisks: *p < 0.05, **p < 0.01, ***p < 0.001.

## 3 Results

### 3.1 Establishment and validation of LCN2 knockout MDA-MB-231 cells

Following the recommendations for cell-based studies (Weiskirchen et al., 2023), we initially conducted an STR profile analysis to confirm the authenticity of the cell lines MDA-MB-231 and MDA-MB-468 used as starting material in our study. Similarity analysis using the CLASTR tool demonstrated a high concordance (>80%) with previously reported STR profiles for both cell lines (Supplementary Table S3; Supplementary Figures S1 and S2). The STR profile of MDA-MB-468 cells showed complete agreement with the reported profile (Yu et al., 2015), while MDA-MB-231 cells displayed only minor variation in one STR marker compared with published data (Yu et al., 2015; Cellosaurus cell line database, 2025; Lorenzi et al., 2009; Fang et al., 2011). Together, these results confirmed the authenticity of both parental cell lines.

To investigate the role of LCN2 in TNBC cells, an LCN2-deficient MDA-MB-231 cell model was generated using CRISPR/Cas9 technology (Figure 1A). Following selection, the bulk pool of transfected MDA-MB-231 cells was tested for successful LCN2KO (Figure 1B). Previous experiments showed that IL-1β induced LCN2 expression in MDA-MB-231 cells (Supplementary Figure S3). Western blot analysis revealed that both targeting approaches 1 and 3 (designated LCN2KO1 and LCN2KO3) showed neither basal nor IL-1β-induced LCN2 expression, indicating a functional knockout. Insertion of the donor DNA was verified, as cells expressed turboGFP (tGFP). LCN2KO2 showed inducible LCN2 expression upon stimulation with IL-1β, but also lower tGFP expression compared with LCN2KO1 and LCN2KO3, suggesting a heterozygous LCN2KO (Figure 1B). After single-cell isolation, all established clones were tested for basal levels of LCN2 by Western blot (Supplementary Figure S4) and RT-qPCR (Supplementary Figure S5). For further analyses, clones #7, #12, and #15 from LCN2KO3 were used (Figure 1C). Selection of these clones was based on their confirmed knockout genotype and was not guided by phenotypic characteristics, thereby minimizing potential selection bias. LCN2 expression has been shown to be induced by different pro-inflammatory stimuli *in vitro* in other cell lines (Schröder et al., 2022; Krizanac et al., 2023). To determine whether LCN2 expression could be induced by additional pro-inflammatory stimuli, parental MDA-MB-231 cells and clone #15 were exposed to selected cytokines (Figure 1D). The highest LCN2 induction was observed after stimulation with IL-1β, followed by TNF-α, in parental MDA-MB-231 cells. No basal expression or induction was observed in clone #15 after stimulation.

**FIGURE 1:**
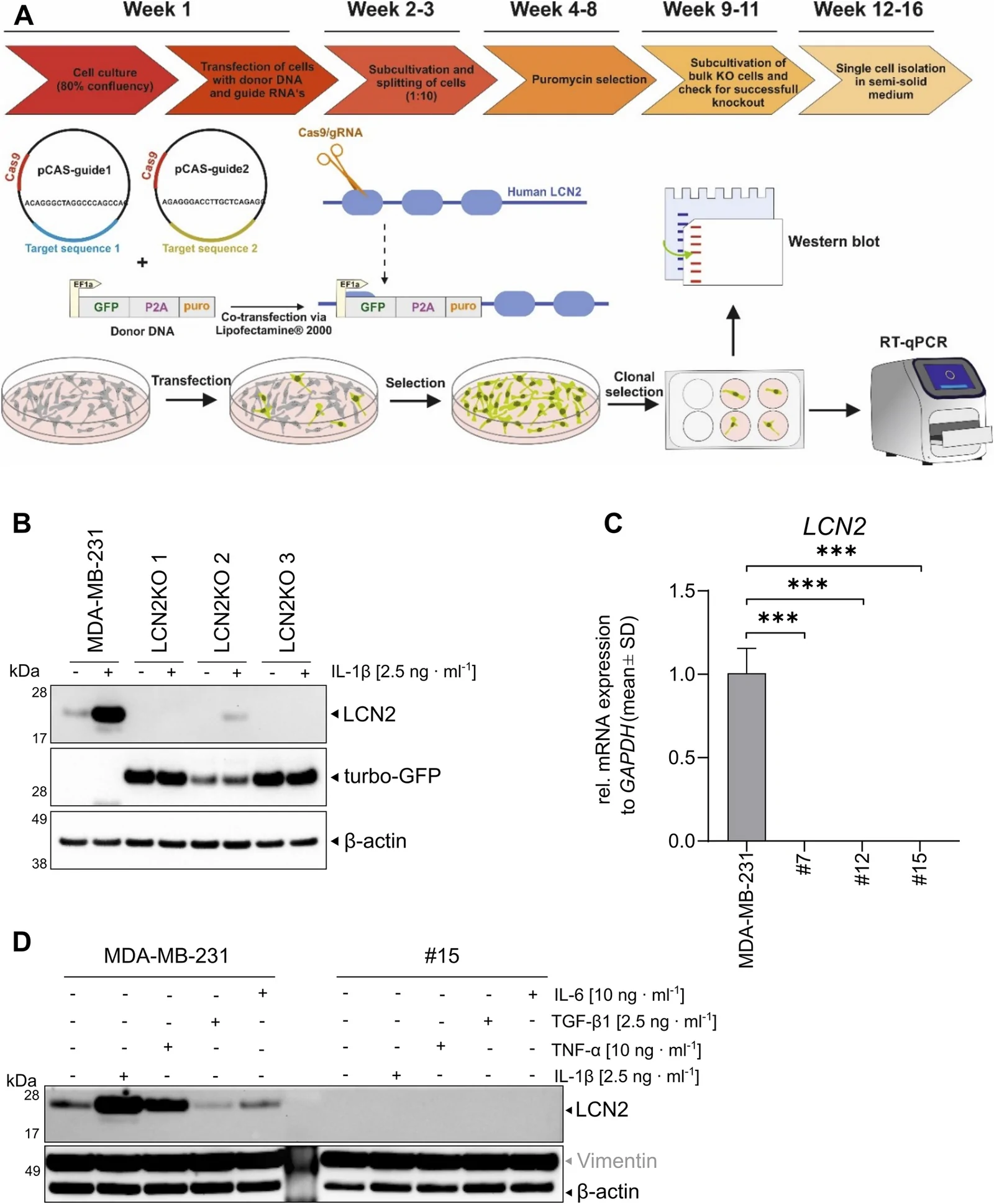
Generation and Validation of LCN2KO MDA-MB-231 Cells. **(A)** Schematic representation of the generation of LCN2KO MDA-MB-231 cells. MDA-MB-231 cells were grown to 80% confluence and co-transfected with donor DNA and two guide RNAs in week 1. Transfected cells were sub-cultivated and split 1:10 in weeks 2–3. Successfully transfected cells were monitored for turbo-GFP expression (green cells). Cells were selected using puromycin for 4–8 weeks until they fully recovered (bulk LCN2 knockout cells). Western blot analysis and RT-qPCR analysis were performed to confirm the successful knockout of LCN2. To generate single-cell clones, bulk cells were cultivated in semi-solid medium in weeks 12–16 and single colonies originating from one cell were picked for sub-cultivation. **(B)** Western blot analysis of bulk LCN2KO1-3 cells treated with 2.5 ng/mL IL-1β. Equal protein loading was confirmed by β-actin (n = 1). Original uncropped blots are presented in WB1 in the Supplementary Material. **(C)** Basal mRNA expression of LCN2 in parental MDA-MB-231 cells compared with single-cell knockout clones #7, #12, and #15. Data is presented as the mean ± SD. Samples were collected from three independent time points (n = 3). Normality was assessed using the Shapiro–Wilk test, and statistical significance was determined by ordinary one-way ANOVA. Statistical significance is denoted with asterisks, ***p < 0.001. **(D)** Western blot of single-cell knockout clone #15 treated with pro-inflammatory cytokines (2.5 ng/mL IL-1β, 10 ng/mL TNF-α, 2.5 ng/mL TGF-β1, or 10 ng/mL IL-6, n = 3). Equal protein loading was confirmed by β-actin. Vimentin was additionally probed on the same membrane (band visible) but is not shown separately. Original uncropped blots are presented in WB2 in the Supplementary Material.

Multicolor fluorescence *in situ* hybridization (M-FISH) analysis was performed to compare the chromosomal profiles of parental MDA-MB-231 cells and LCN2KO #15 cells and to assess whether the generation of the CRISPR/Cas9-edited LCN2 knockout clone was associated with alterations in chromosome number or structural chromosomal abnormalities (Figure 2). As expected for this highly aneuploid cell line, parental MDA-MB-231 cells exhibited a complex karyotype with multiple numerical and structural chromosomal abnormalities. LCN2KO #15 cells retained the major chromosomal characteristics of the parental cell line, including the main structural rearrangements, without evidence of additional large-scale chromosomal alterations following genome editing. The detailed chromosomal abnormalities identified in parental and LCN2KO #15 cells are summarized in Table 1. LCN2KO #15 was derived from a defined parental subclone, resulting in a more homogeneous chromosomal profile compared with the heterogeneous parental population. Minor differences observed between parental and LCN2KO #15 cells were consistent with subclonal selection rather than genome-editing-associated genomic instability.

**FIGURE 2:**
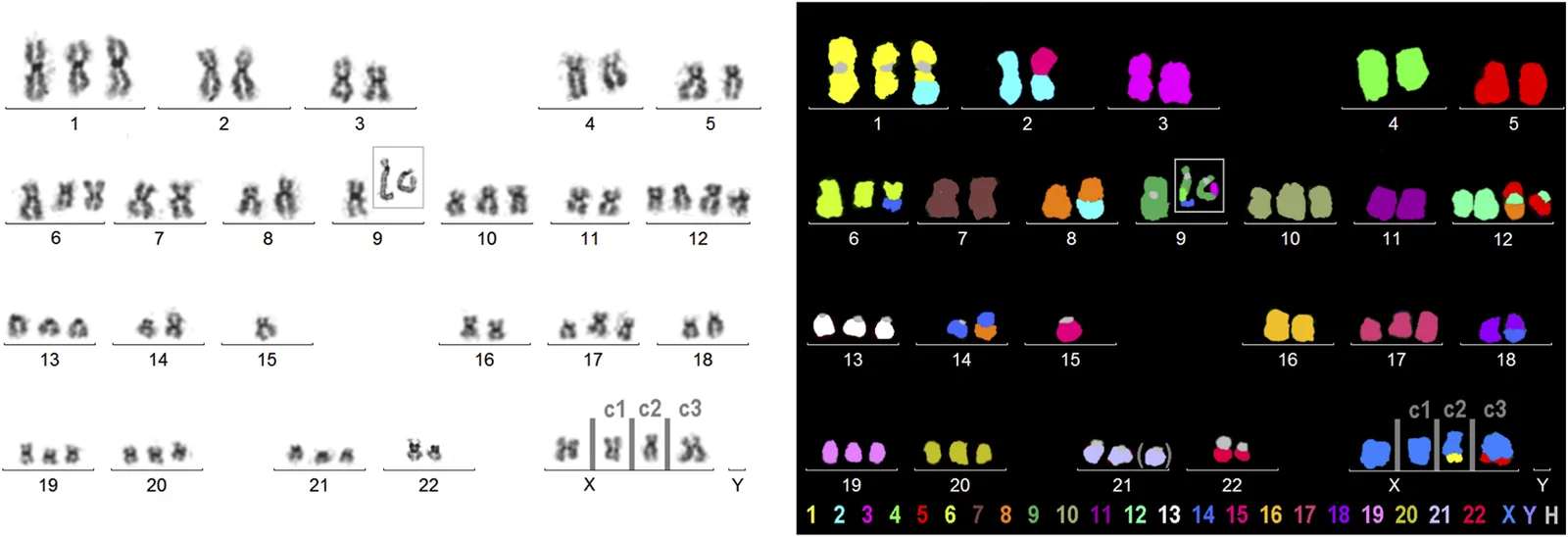
Multicolor FISH Analysis of Parental MDA-MB-231 Cells. Representative metaphase spreads of parental MDA-MB-231 cells following multicolor fluorescence *in situ* hybridization (M-FISH) are shown. Individual chromosomes are displayed in distinct pseudo-colors based on chromosome-specific probe combinations, enabling the identification of structural and numerical chromosomal aberrations. The parental cell line exhibits a highly complex and aneuploid karyotype with multiple derivative chromosomes and translocations. All chromosomes shown are from one metaphase, apart from derivative chromosomes 9 (marked by a gray square) and the derivative X-chromosomes highlighted as clones c2 and c3. The derivative chromosomes 9 were present but not well spread and resolved in the metaphase shown; thus, the corresponding chromosomes from another, well-spread but incomplete second metaphase are depicted instead. There are three (about equally sized) clones in the MDA-MB-231 cell line with respect to the second X-chromosome: clone 1 (c1) has a shortened X-chromosome in the long arm, del(X)(q2?6). The other two clones (c2 and c3) have a derivative X, each with parts of chromosomes 1 or 5 at the end of the shortened long arm. Chromosome 21 was present two or three times in all three clones. Clones c1 and c2 were associated with the dic(2;15)(p11;q11), whereas clone c3 was associated with the der(2)t(2;9)(q2?2;?).

**TABLE 1:** Composite karyotype of parental and LCN2KO #15.

Parental MDA-MB-231	LCN2KO #15
del(X)(q2?6) or der(X)t(X;1)(q2?6;?) or der(X)t(X;5)(q2?6;?)	del(X)(q2?6)
+der(1)t(1;2)(q2?5;p2?2)	+der(1)t(1;2)(q2?5;p2?2)
dic(2;15)(p11;q11) or der(2)t(2;9)(q2?2;?)	dic(2;15)(p11;q11)
+del(3)(p1?3)	+del(3)(p1?3)
del(6)(q1?2) +der(6)t(6;14)(q1?2;q1?)	del(6)(q1?2) +der(6)t(6;14)(q1?2;q1?)
der(8)t(2;8)(p1?2;q1?2)	der(8)t(2;8)(p1?2;q1?2)
der(9)t(3;9)(?;q3?4) +der(9)(9pter->9q3?4::4?->4?::14q?->14qter)	der(9)t(3;9)(?;q3?4) +der(9)(9pter->9q3?4::4?->4?::14q?->14qter)
+10	+10
+der(12)(8qter->8q2?1::12p1 or 12q1->12p1 or 12q1::5p1?1.2->5pter) +der(12)(:12q1->12p1 or 12q1::5p1?1.2->5pter)	+der(12)(8qter->8q2?1::12p1 or 12q1->12p1 or 12q1::5p1?1.2->5pter) +der(12)(:12q1->12p1 or 12q1::5p1?1.2->5pter)
+13	+13
del(14)(q11.2),der(14)t(8;14)(?;p11.1)	del(14)(q11.2),der(14)t(8;14)(?;p11.1)
−15	−15
+17	+17
der(18)t(14;18)(q?;p1?1)	der(18)t(14;18)(q?;p1?1)
+19	+19
+20	+20
mos +21 and no +21	No +21

Collectively, these findings demonstrate that we successfully established and validated a functional LCN2 knockout in MDA-MB-231 cells. The generated knockout model preserves the complex genomic background of the parental MDA-MB-231 cell line while representing a more homogeneous subpopulation. This confirms its suitability for downstream functional and molecular analyses, in which observed phenotypic differences can be attributed primarily to the targeted disruption of LCN2 rather than to unintended karyotypic alterations.

### 3.2 Morphological and phenotypic characteristics of LCN2 knockout MDA-MB-231 cells

To characterize the cellular phenotype associated with LCN2 loss, we compared the morphological and phenotypic features of parental MDA-MB-231 cells and LCN2KO clones. These analyses included light and fluorescence microscopy, transmission electron microscopy, and assessment of epithelial and mesenchymal marker expression.

Light and fluorescence microscopic analyses were conducted to evaluate cellular morphology. Parental MDA-MB-231 cells exhibited an elongated, spindle-shaped morphology when they were not fully confluent (Supplementary Figure S6). These cells grew in an adherent monolayer and did not form compact clusters, instead growing individually with frequent overlapping, resulting in a disorganized growth pattern. #15 cells largely maintained these morphological characteristics but also showed a tendency to form enlarged cells (Supplementary Figure S7). The actin cytoskeleton plays a crucial role in cell shape, movement, and internal organization (Stricker et al., 2010). F-actin was visualized using rhodamine-conjugated phalloidin. Counterstaining with DAPI revealed oval-shaped nuclei in both cell lines (Figures 3A,B). Enlarged LCN2KO#15 cells displayed F-actin stress fibers, together with either multiple nuclei (white arrows) or enlarged nuclei (white asterisk, Figure 3B).

**FIGURE 3:**
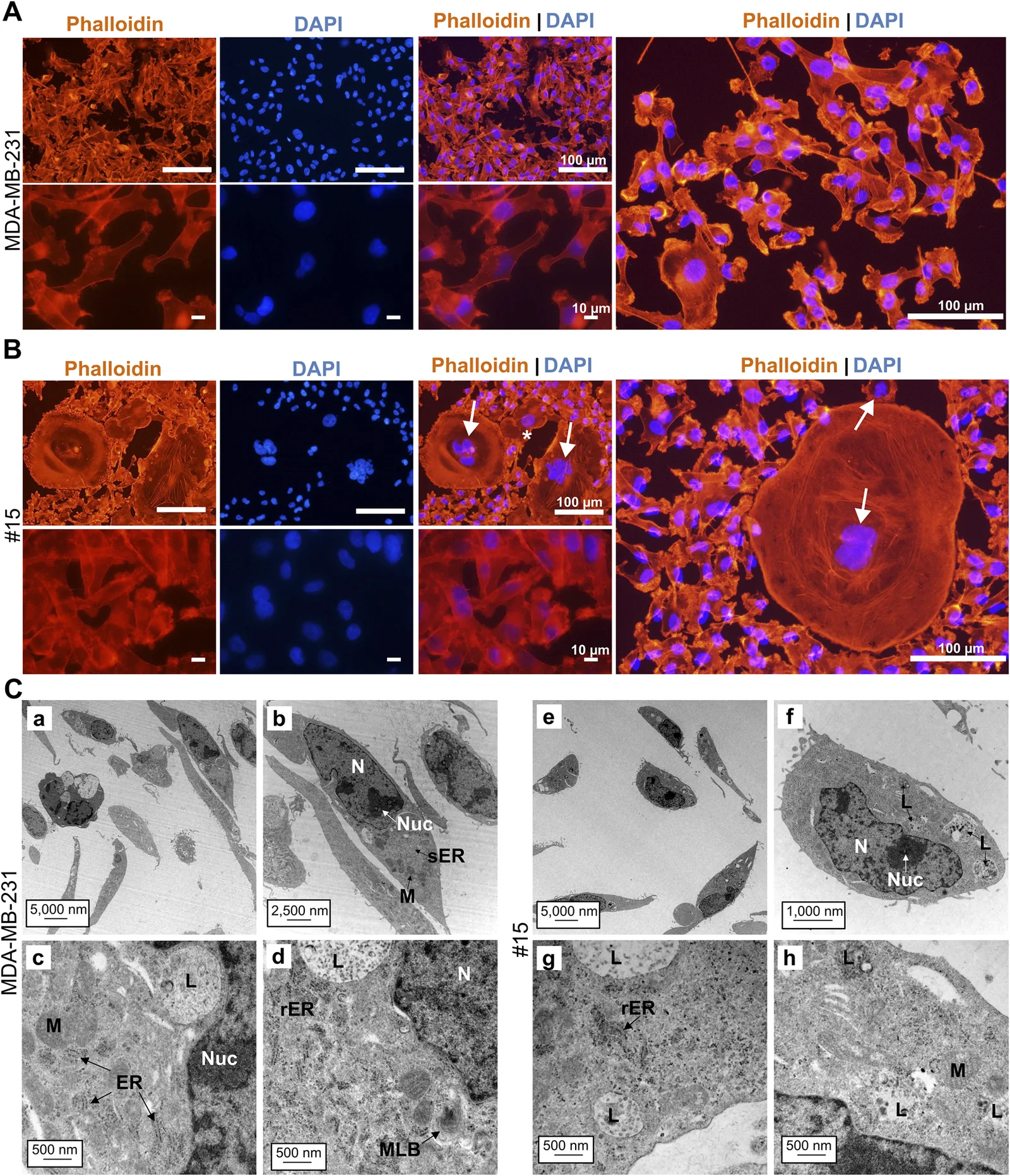
Phenotypic Analysis of Parental MDA-MB-231 and LCN2KO #15 Cells. Fluorescence microscopy images of parental MDA-MB-231 **(A)** and LCN2KO clone #15 **(B)** are shown. The F-Actin cytoskeleton of parental MDA-MB-231 and LCN2KO #15 was stained using rhodamine phalloidin (orange) and counterstained with DAPI (blue). Scale bars represent 10 µm or 100 μm, as indicated. Images were captured at 100×, 200× and 600× magnifications. Arrows indicate multinucleated enlarged cells, and asterisks label cells with enlarged nuclei **(C)** Ultrastructure analysis by transmission electron microscopy of parental MDA-MB-231 **(a–d)** and LCN2KO #15 **(e–h)** cells. Parental MDA-MB-231 **(a)** and LCN2KO #15 **(e)** display a spindle-like, elongated morphology. Both cell lines have a prominent nucleus (n) with visible nucleoli (Nuc) **(b,f)**. At higher magnifications, organelles such as mitochondria (M), endoplasmic reticulum (rough ER, smooth ER), myeloid bodies (MLB), and lysosomes (L) are visible **(c,d,g,h)**. Images were taken at magnifications of 1,670× **(a,e)**, 3,597× **(b)**, 6,000× **(f)**, 16,700× **(d)**, and 21,560× **(c,g,h)**. Scale bars represent 500 nm, 1,000 nm, 2,500 nm, and 5,000 nm, as indicated.

Subcellular organization cannot be resolved using optical microscopy. Therefore, ultrastructural analysis was performed by transmission electron microscopy (TEM, Figure 3C). Consistent with the light microscopic images, parental MDA-MB-231 and LCN2KO #15 cells exhibited an elongated morphology (Figure 3C a,e). Both cell lines had a prominent nucleus with abundant euchromatin and relatively sparse electron-dense heterochromatin, indicative of high transcriptional activity and metabolically active cells (Figure 3C b,f). The nuclei displayed clearly defined subnuclear regions, including distinct nucleoli. At the cell periphery, mitochondria appeared as oval to elongated organelles of variable size and shape and were characterized by tubular cristae (Figure 3C d,h). Myeloid bodies primarily function in the storage, degradation, and processing of lipids and other cellular materials (Brok et al., 2018). They appeared round or oval and were composed of whorled layers of lipid-rich or proteinaceous material. The layers were electron-dense, giving them an onion-like appearance (Figure 3C d). Furthermore, rough endoplasmic reticulum (rER) appeared as a network of flattened and membrane-bound cisternae. The cytoplasmic surface was studded with ribosomes, giving it a rough, granular appearance (Figure 3C c,d,g). Lysosomes appeared as spherical membrane-bound organelles. Their lumen was electron-dense and appeared heterogeneous, with partially digested material giving it a granular appearance (Figure 3C c,d,g,h). LCN2KO #15 cells exhibited slightly more lysosomes than parental MDA-MB-231 cells, while the morphology and abundance of all other organelles were comparable. The higher number of lysosomes in #15 suggests that these cells may have increased lysosomal activity or cellular turnover.

Given that MDA-MB-231 cells exhibit a characteristic mesenchymal phenotype, we next examined the expression of mesenchymal and epithelial markers in parental and LCN2KO cells to further characterize the cellular phenotype (Figure 4). Western blot analysis showed sustained expression of the mesenchymal markers fibronectin 1 (FN1), α-smooth muscle actin (α-SMA) and vimentin in parental MDA-MB-231 cells as well as in the LCN2KO clones #7, #12, and #15 (Figure 4A). In contrast, the epithelial marker keratin 19 was only weakly expressed. E-cadherin was only weakly expressed in parental MDA-MB-231 cells, whereas the KO clones showed undetectable levels. These findings were consistent with RT-qPCR analysis, which showed downregulation of *CDH1* in the LCN2KO clones (Figure 4B). *VIM* expression remained unchanged between parental cells and LCN2KO clones, whereas *FN1* expression was increased in clones #7 and #15. In contrast, *ACTA2* expression was reduced in LCN2KO #15.

**FIGURE 4:**
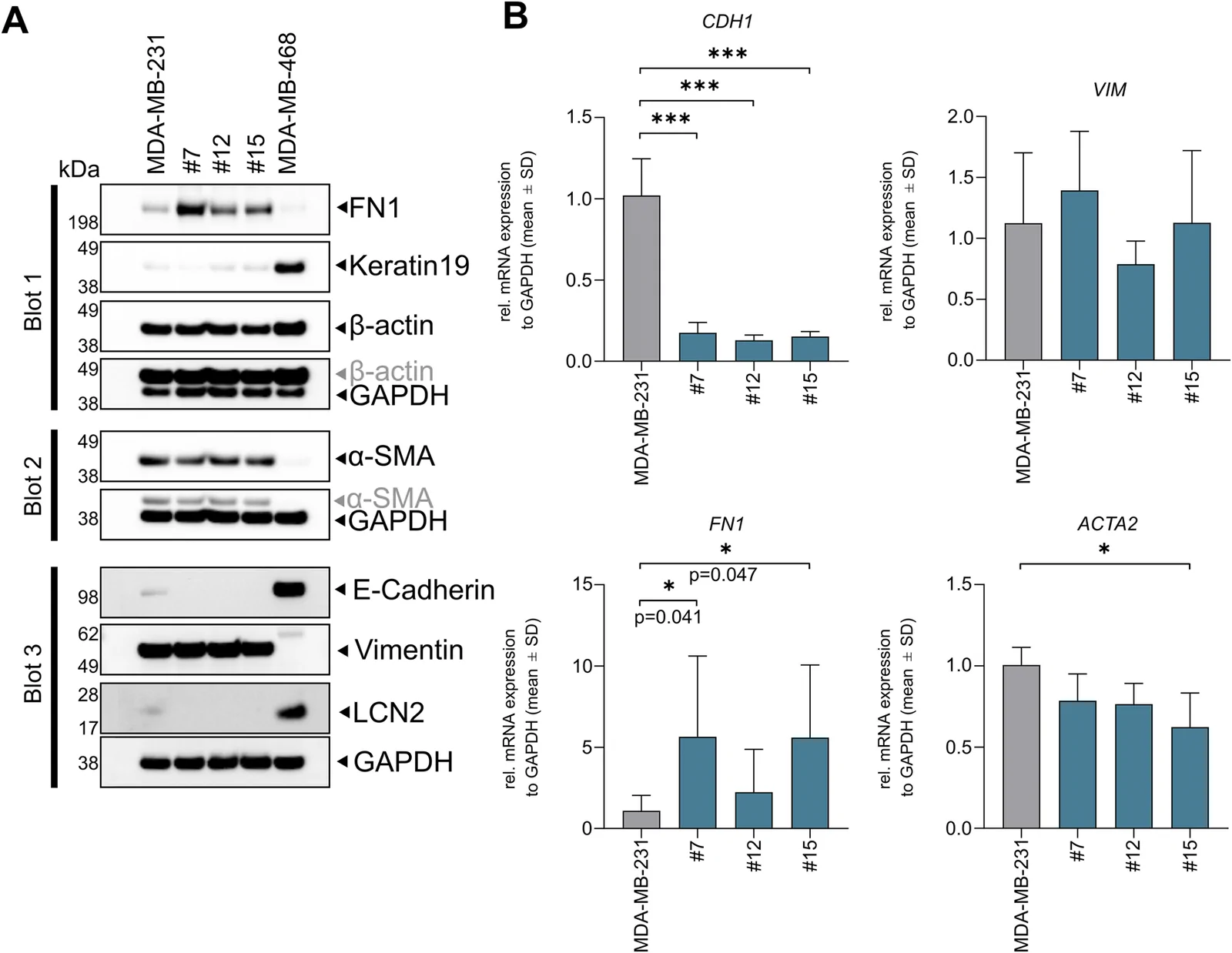
Expression of Mesenchymal and Epithelial Markers. **(A)** Representative Western blots of protein extracts from parental MDA-MB-231 cells, KO clones #7, #12, and #15, and MDA-MB-468 cells, an epithelial TNBC cell line used as a control for epithelial marker expression. Equal protein loading was confirmed by β-actin and GAPDH. Because several target proteins had overlapping molecular weights, the marker panel was split across three separate membranes, as indicated (Blot 1–3). Original uncropped blots are presented in WB3-5 in the Supplementary Material. **(B)** Relative mRNA expression of epithelial and mesenchymal marker genes *CDH1*, *VIM, FN1,* and *ACTA2* in parental MDA-MB-231 cells and KO clones #7, #12, and #15. All experiments were conducted in three independent replicates (n = 3). Data is presented as the mean ± SD. Normality was assessed using the Shapiro–Wilk test, and statistical significance was determined by ordinary one-way ANOVA with Dunnett’s multiple comparisons test or the Kruskal–Wallis test with Dunn’s multiple comparisons test. Statistical significance is denoted with asterisks: *p < 0.05, **p < 0.01, ***p < 0.001.

Overall, these results demonstrate that LCN2KO MDA-MB-231 cells maintain the mesenchymal identity characteristic of this cell line. These findings provide a phenotypic framework for subsequent transcriptomic analyses of molecular changes associated with LCN2 loss.

### 3.3 Transcriptomic profiling of parental MDA-MB-231 and LCN2KO #15 by NGS data

To obtain an initial overview of the transcriptional consequences of LCN2 loss, NGS transcriptomic profiling was performed using LCN2KO clone #15, a confirmed complete LCN2 knockout clone. This analysis was intended to characterize the transcriptional landscape associated with LCN2 loss in an individual knockout clone and to identify candidate pathways and molecular changes for further investigation. Importantly, the NGS findings were considered clone-specific observations until confirmed in additional independent LCN2 knockout clones. An overview of the RNA sequencing dataset is provided in Supplementary Table S4, which includes all detected genes with associated gene identification numbers (gene IDs) and normalized counts (transcripts per million). Differential gene expression was calculated using parental cells as the reference (baseline) group and LCN2KO cells as the comparison group. Therefore, positive log_2_ fold-change (log_2_fc) values indicate genes upregulated in LCN2KO cells relative to parental cells, whereas negative log_2_fc values indicate downregulated genes in LCN2KO cells.

To assess the overall transcriptional similarity and variability among the RNA-seq samples, principal component analysis (PCA) was performed. PC1 and PC2 accounted for 78.77% and 9.44% of the total variance, respectively, together explaining 88.21% of the overall transcriptional variance (Figure 5A). The LCN2KO replicates (LCN2KO1-3) clustered closely with each other and were clearly separated from the parental samples (MDA-MB-231wt_1-3) primarily along PC1, indicating distinct global transcriptional profiles between the two groups. The parental replicates showed greater dispersion, with samples 1 and 3 clustering more closely, whereas sample two was separated from the other parental samples mainly along PC2. Notably, MDA-MB-231wt_2 did not exhibit any apparent deviation from the standard RNA-seq quality-control parameters, including STAR alignment metrics (data not shown). To initially define the global transcriptional consequences of LCN2 loss without restricting the analysis to predefined biological pathways, we performed unbiased pathway enrichment analyses of the LCN2KO #15 transcriptome. Gene set enrichment analysis (GSEA) and Gene Ontology (GO) analyses were used to identify biological processes and signaling pathways broadly affected by LCN2 loss, providing a basis for subsequent targeted analyses of mesenchymal/EMT, iron/ferroptosis, and inflammatory signaling pathways. The GSEA ridge plot revealed positive enrichment of innate immune and inflammatory pathways among genes upregulated in LCN2KO #15, including chemokine response, cytokine-mediated signaling, and bacterial response pathways (Figures 5B–D). Consistent with these findings, Gene Ontology (GO) analysis of upregulated genes identified enrichment for ribosome biogenesis, rRNA processing, translational pathways, and canonical NF-κΒ signaling (Figure 5E). In contrast, genes downregulated in LCN2KO #15 cells were primarily associated with mitochondrial organization, metabolic pathways, ECM organization, and protein trafficking (Figure 5F). Together, these enrichment analyses revealed gene sets and pathways that were differentially represented in LCN2-deficient cells, including those related to immune signaling, biosynthetic processes, cellular metabolism, and structural organization.

**FIGURE 5:**
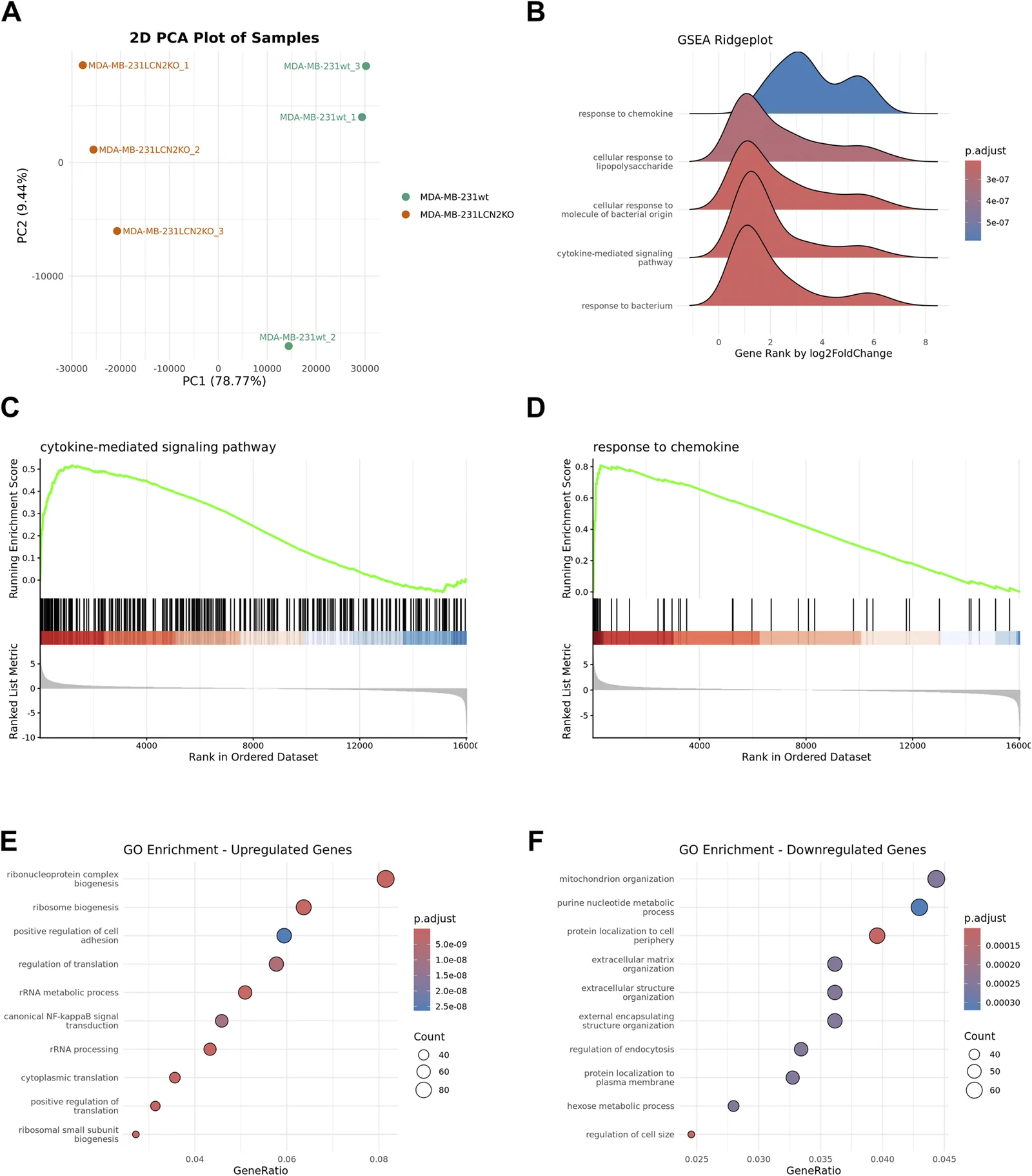
Gene Set Enrichment Analysis (GSEA) and Gene Ontology (GO) Enrichment of Differential Gene Expression Between Parental MDA-MB-231 and LCN2KO #15 Cells. RNA-seq data were used to compare parental MDA-MB-231 and LCN2 knockout clone #15. **(A)** Principal component analysis (PCA) was performed on the RNA-seq expression data generated from parental MDA-MB-231 cells (MDA-MB-231wt1-MDA-MB-231wt3) and LCN2KO clone #15 (KO1–KO3). **(B)** The ridge plot shows significantly enriched biological processes based on ranked genes ordered by log_2_ fold change. **(C,D)** Representative GSEA enrichment plots illustrate positive enrichment for the cytokine-mediated signaling pathway and response to chemokine, with enrichment scores indicating that genes associated with these pathways are predominantly upregulated in knockout clone #15. Vertical bars indicate the position of pathway genes within the ranked gene list. **(E)** GO enrichment analysis of significantly upregulated genes reveals enrichment of pathways related to ribosome biogenesis, rRNA processing, translation, and NF-κB signaling. **(F)** GO enrichment analysis of downregulated genes shows enrichment for processes associated with mitochondrial organization, extracellular matrix organization, nucleotide metabolism, and membrane protein localization. Dot size represents the number of genes contributing to each term, while color indicates the adjusted p-value (p.adjust).

#### 3.3.1 Transcriptomic characteristics associated with the mesenchymal phenotype

To further characterize the cellular phenotype following LCN2 loss, we examined the expression of established mesenchymal and epithelial markers. This analysis was performed to determine whether the transcriptomic changes were consistent with the mesenchymal phenotype previously characterized by Western blot and RT-qPCR analyses. We therefore hypothesized that the previously observed phenotypic characteristics of the LCN2 knockout cells would be reflected at the transcriptomic level by corresponding changes in mesenchymal and epithelial marker expression. Consistent with these observations, transcriptomic analysis showed that key mesenchymal-associated genes, including *VIM* and *CDH2*, remained largely unchanged in LCN2KO #15 cells, whereas *FN1* and *ITGA5* were upregulated (Supplementary Table S5). Although *ACTA2* showed a non-significant decrease, the overall expression pattern did not indicate a broad transcriptional shift away from the mesenchymal phenotype. Similarly, epithelial-associated genes did not show evidence of a coordinated MET following LCN2 depletion. Although the expression levels of *CDH1, KRT8,* and *KRT18* were reduced, *EPCAM, CLDN1,* and *OCLN* showed unchanged or increased expression patterns, suggesting the absence of a consistent epithelial gene signature.

Analysis of genes associated with cellular plasticity and hybrid EMT states revealed mixed changes, including reduced *AXL* expression and increased *CD44* expression, while *TGFBR1* and *TGFBR2* expression remained unchanged (Supplementary Table S5). Together, these findings suggest that LCN2 deficiency is associated with changes in selected EMT-related transcripts but does not result in a pronounced alteration of the mesenchymal/hybrid phenotype characteristic of MDA-MB-231 cells.

#### 3.3.2 Alterations in iron homeostasis, oxidative stress and ferroptosis-related pathways

The unbiased pathway enrichment analyses identified differential expression of genes associated with iron homeostasis, prompting a focused analysis of this pathway. Because cellular iron availability is closely linked to redox balance, lipid peroxidation, and ferroptotic susceptibility, we further examined genes involved in iron handling and iron-dependent cell death. We hypothesized that LCN2 loss may alter the expression of iron homeostasis-related genes, potentially linking changes in iron handling to oxidative stress and ferroptosis-related processes (Flo et al., 2004).

Analysis of iron metabolism-associated genes revealed increased expression of *TFRC*, *FTH1*, and *FTL* in LCN2KO cells, whereas *SLC40A1* remained unchanged (Supplementary Table S5). Among ferroptosis-related genes, *SLC7A11* was upregulated, while the expression of *GPX4* and *ACSL4* was reduced, with no significant change in *NCOA4*. These expression patterns suggest that LCN2 deficiency is associated with transcriptional changes in genes involved in iron handling and ferroptosis-related processes.

Oxidative stress-associated genes also showed selective changes following LCN2 loss. Although *NFE2L2/NRF2* expression remained unchanged, increased expression of the NRF2 target genes *NQO1* and *GCLM* was observed. In addition, *KEAP1* expression was increased, while *HMOX1* remained unchanged. A shift in antioxidant-related gene expression was further suggested by increased mitochondrial *SOD2* and reduced *SOD1* expression. Together, these findings indicate that LCN2 deficiency is associated with altered expression of selected genes involved in iron metabolism, ferroptosis regulation, and oxidative stress responses. Further functional studies will be required to determine whether these transcriptional changes correspond to altered iron metabolism or redox homeostasis.

#### 3.3.3 Enrichment of inflammatory and transcriptional regulatory pathways

The unbiased pathway enrichment analyses also revealed differential representation of genes associated with inflammatory and immune-related signaling, prompting a more focused investigation of these pathways. We therefore examined the expression of genes involved in inflammatory signaling to determine whether the transcriptomic changes in LCN2KO #15 cells were consistent with activation of inflammatory responses. We hypothesized that loss of LCN2 may be associated with altered inflammatory signaling, as reflected by changes in the expression of inflammation-related genes.

Assessment of inflammatory and NF-κB-associated genes revealed increased expression of *RELA*, *NFKBIA*, and *NFKBIZ* in LCN2KO #15 cells (Supplementary Table S5). In addition, pro-inflammatory cytokine-associated genes, including *IL1B*, *IL6*, and *CXCL8*, showed increased expression compared with parental cells, with *CXCL8* displaying the most pronounced upregulation. These findings indicate that LCN2 deficiency is associated with changes in the expression of genes involved in inflammatory signaling and NF-κB regulation.

To validate these transcriptomic findings, selected inflammatory genes were analyzed by RT-qPCR. Increased expression of *NFKBIA*, *CASP1*, *CXCL1*, and *IL1B* was confirmed in LCN2KO #15 cells (Supplementary Figure S8A). Consistent with the transcriptomic data, increased basal protein levels of the NF-κB regulators IκBα and IκBζ were observed in LCN2KO cells (Supplementary Figure S8B). Following stimulation with IL-1β or TNF-α, IκBζ induction was observed in both cell lines, confirming the responsiveness of the NF-κB pathway. However, phosphorylation of NF-κB p65 showed stimulus-dependent differences, with reduced induction in LCN2KO #15 cells under several stimulation conditions, whereas total NF-κB levels remained unchanged. Although CXCL8 protein expression was induced after inflammatory stimulation in both cell lines, the marked transcriptional increase observed by RNA-seq was not reflected at the protein level. As expected, LCN2 secretion was induced by IL-1β and TNF-α stimulation in parental cells but was absent in LCN2KO #15 cells.

### 3.4 Effects of LCN2 knockout on cell migration, invasion, and adhesion

Given the observed morphological and molecular differences following LCN2 depletion, we next investigated whether loss of LCN2 affected the migratory capacity of MDA-MB-231 cells. Cell migration was assessed using a Transwell migration assay (Figure 6). MCF-7 cells, which have previously been described as non-migratory (Higgins and Gomillion, 2025), were used as a control (Figure 6A). LCN2KO #7 and #15 cells exhibited significantly reduced migratory capacity compared with parental MDA-MB-231 cells (Figure 6B), suggesting that LCN2 loss is associated with altered migratory behavior. To further examine this association, LCN2 was re-expressed in LCN2KO cells (Supplementary Figure S9A). Rescue experiments showed increased migration following LCN2 re-expression compared with GFP-transfected controls (Supplementary Figure S9B).

**FIGURE 6:**
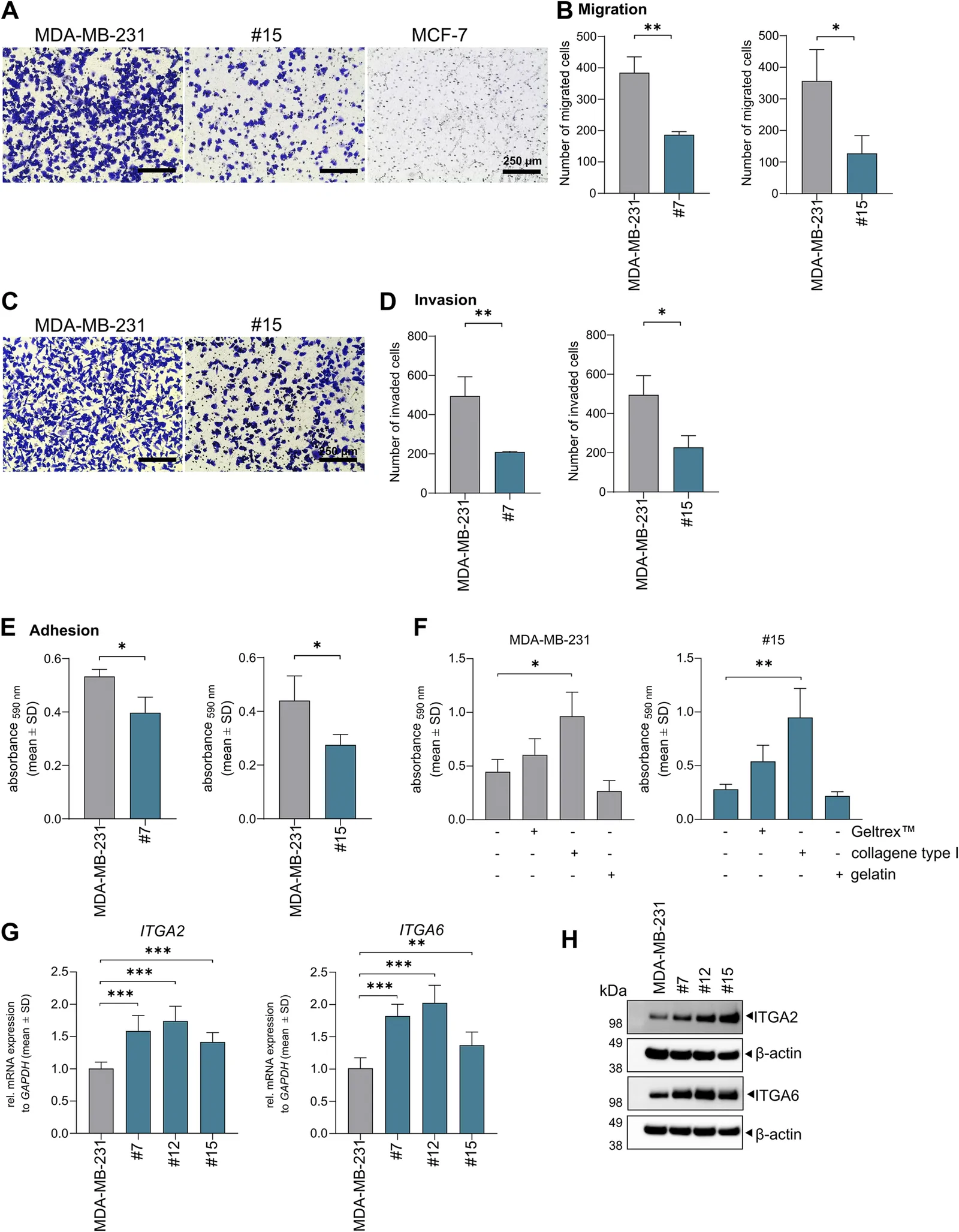
LCN2 Loss Alters Cell Migration, Invasion and Adhesion. **(A)** Representative images of three independent experiments showing migration of parental MDA-MB-231, LCN2KO #15, and MCF-7 cells through hanging inserts with an 8.0 µm pore size. Migrated cells were stained with 0.2% (w/v) crystal violet solution. Ten representative fields of view were imaged per insert for quantification. Scale bars represent 250 µm. MCF-7 cells were used as a negative control. **(B)** Migrated cells from parental MDA-MB-231 cells and LCN2KO clones #7 and #15 were quantified using ImageJ (1.54p). Data is shown as the mean ± SD (n = 3). **(C)** Representative images of three independent experiments showing invasion of parental MDA-MB-231 and LCN2KO #15 through hanging inserts with an 8.0 µm pore size. Invaded cells were stained with 0.2% (w/v) crystal violet solution. Scale bars represent 250 µm. **(D)** Invaded cells from parental MDA-MB-231 cells and LCN2KO clones #7 and #15 were quantified using ImageJ (1.54p). Data is shown as the mean ± SD (n = 3). **(E)** Parental MDA-MB-231 cells and LCN2 knockout clones #7 and #15 were used for adhesion assay. Cell lines were seeded in 48-well cell culture plates in triplicate. Following incubation, adherent cells were stained with 0.2% (w/v) crystal violet. The bound dye was solubilized, and absorbance was measured at 590 nm as an indicator of cell adhesion. Data is shown as the mean ± SD (n = 3 for #7 and n = 4 for #15). **(F)** Cell culture plates were coated with 0.1% Geltrex, 50 μg/mL rat tail collagen I, or 0.1% gelatin prior to cell seeding. Adherent cells were stained with 0.2% (w/v) crystal violet solution, and the absorbance of solubilized dye was measured at 590 nm. Data is shown as mean ± SD (n = 3). **(G)** Relative mRNA expression levels of *ITGA2* and *ITGA6* were determined in parental MDA-MB-231 cells and LCN2KO clones #7, #12, and #15. Data is shown as the mean ± SD (n = 3). **(H)** Representative Western blot showing protein expression of ITGA2 and ITGA6 in parental MDA-MB-231 cells and LCN2 knockout clones #7, #12, and #15. β-Actin served as the loading control (n = 3). Original uncropped blots are presented in WB6 in the Supplementary Material. Normality was assessed using the Shapiro–Wilk test, and statistical significance was determined by either a *t-*test **(B,D,E)** or ordinary one-way ANOVA with Dunnett’s multiple comparisons test or the Kruskal-Wallis test with Dunn’s multiple comparisons test **(F,G)**. Statistical significance is indicated by asterisks, *p < 0.05, **p < 0.01, ***p < 0.001.

To determine whether the effects of LCN2 depletion on cell motility extended to invasive behavior, we next assessed the invasive capacity of MDA-MB-231 cells using a Transwell invasion assay (Figures 6C,D). Similarly, LCN2KO #7 and #15 cells exhibited reduced invasive capacity compared with parental MDA-MB-231 cells. Re-expression of LCN2 in LCN2KO #15 cells showed partially restored invasion compared with the GFP-transfected control (Supplementary Figure S9C).

Because cell adhesion to extracellular matrix components is an important determinant of tumor cell migration and invasion, adhesive properties were subsequently assessed (Figure 6E). LCN2KO #7 and #15 cells displayed reduced adhesion to uncoated culture surfaces compared with parental cells. Re-expression of LCN2 showed a trend toward increased adhesion in LCN2KO cells (Supplementary Figure S9D). To investigate whether extracellular matrix composition influenced adhesion, cells were seeded on plates coated with Geltrex, collagen type I, or gelatin (Figure 6F). Both parental and LCN2KO #15 cells showed increased adhesion to collagen type I, with LCN2KO #15 reaching adhesion levels comparable to parental cells. Geltrex coating also enhanced adhesion of LCN2KO #15 cells. To explore potential molecular mechanisms underlying these adhesion differences, the expression of the ECM receptors *ITGA2* and *ITGA6* was analyzed. Both transcript and protein levels were increased in LCN2KO cells compared with parental cells (Figures 6G,H). These findings indicate that LCN2 deficiency is associated with altered expression of integrin receptors involved in cell–matrix interactions. Whether the increased expression of ITGA2 and ITGA6 functionally contributes to the differential adhesion response to specific extracellular matrix components remains to be determined. Cell proliferation was not affected by LCN2 deficiency (Supplementary Figure S10).

Overall, the analysis of LCN2KO MDA-MB-231 single-cell-derived clones revealed changes in cellular morphology and marker expression, together with reduced migration and altered adhesion properties. Collectively, these findings suggest that LCN2 may contribute to the regulation of cellular properties involved in migration and interaction with the extracellular matrix in MDA-MB-231 cells.

## 4 Discussion

TNBC remains one of the most challenging breast cancer subtypes, lacking targeted therapies. Therefore, it is crucial to identify molecular drivers, discover biomarkers, and reduce treatment toxicity and resistance (Schwentner et al., 2012). This study demonstrates that loss of LCN2 results in altered cell architecture, impaired cellular organization, and altered signaling. Simultaneous suppression of ECM assembly, remodeling, and cell-matrix adhesion indicates decreased structural integrity and weakened interactions with the surrounding microenvironment. Functional analysis further supports these findings, as cell adhesion and migration were reduced upon LCN2 knockout.

MDA-MB-231 cells were initially isolated from the pleural effusion of a 51-year-old Caucasian female with metastatic breast adenocarcinoma in 1973 (Amaro et al., 2016). These cells are characterized by a mesenchymal phenotype (Xiao et al., 2021; Fenizia et al., 2019), supported by their morphology and expression of mesenchymal markers such as vimentin (Wei et al., 2018) and the absence of E-cadherin (Xiao et al., 2021). Over the years, MDA-MB-231 cells have become a commonly used cell line model in TNBC research. In the search for biomarkers, attention has turned to LCN2, which shows increased expression in metastatic breast cancer (Yang et al., 2009). LCN2 has been linked to promoting proliferation, migration, and invasion, and to suppressing apoptosis, thereby prolonging cancer cell lifespan (Bao et al., 2024). Despite evidence of the role of LCN2 in tumor progression and metastasis in TNBC, its precise molecular mechanisms remain unclear. Additionally, its potential context-dependent role across TNBC subtypes and its contribution to chemoresistance require further investigation.

In this study, successful functional knockout of LCN2 was confirmed by RT-qPCR and Western blot analyses, demonstrating the complete loss of intact *LCN2* mRNA spanning the edited region and the absence of functional LCN2 protein under both basal conditions and following stimulation with pro-inflammatory cytokines. Interestingly, RNA-seq still detected reads mapping to the *LCN2* locus in knockout cells, despite the lack of detectable protein. This discrepancy is likely explained by the CRISPR/Cas9 HDR-mediated knockout strategy, which disrupts the coding sequence through insertion of a GFP-puromycin cassette while leaving the endogenous promoter intact (NGAL (LCN2) Human Gene Knockout Kit (CRISPR), #KN207685, OriGene Technologies Inc., Rockville, MD, United States of America). Consequently, transcription from the *LCN2* locus may still occur, generating disrupted or aberrantly processed transcripts that are detected by RNA-seq but are incapable of producing functional protein. Furthermore, because transcript quantification was performed using the human reference transcriptome, reads originating from the edited allele may still be assigned to *LCN2*. In contrast, the RT-qPCR primers were designed to span the edited region and therefore specifically detect only the intact wild-type transcript. These findings highlight that transcript abundance does not necessarily reflect the production of functional protein in CRISPR-edited cells and emphasize the importance of validating gene disruption at both the RNA and protein levels.

To further investigate the cellular consequences of LCN2 loss, morphological analysis of LCN2KO cells revealed alterations in cellular architecture. Compared with parental cells, LCN2KO cells displayed an increased incidence of enlarged cells. This enlarged morphology in a subset of LCN2KO cells may be compatible with senescence-like features or a senescence-associated secretory phenotype (SASP) (Gu and Kitamura, 2012). However, we did not evaluate established markers of cellular senescence, such as senescence-associated β-galactosidase (SA-β-gal) activity, p16, p21, γH2AX, or the secretion of SASP-associated cytokines in the present study (Gu and Kitamura, 2012; Wang et al., 2024). Therefore, these observations should be interpreted with caution. Future studies are needed to determine whether LCN2 deficiency promotes cellular senescence by comprehensively assessing canonical senescence markers, characterizing the associated secretory phenotype, and elucidating the underlying molecular mechanisms. Furthermore, this morphology is commonly associated with defects in mitotic progression, cytokinesis, or cell cycle regulation (Chang et al., 2025; Söllner et al., 2002), suggesting a previously unrecognized role for LCN2 in maintaining cell division fidelity. Importantly, the formation of enlarged cells was consistently observed not only in TNBC cells but also in an independent cancer cell model following LCN2KO, indicating that this phenotype is not restricted to a single cellular background but may represent a broader consequence of LCN2 depletion in malignant cells (Gäberlein et al., 2026). Transcriptomic analysis further supports a structural and mechanistic basis for these morphological alterations. GO enrichment analysis of downregulated transcripts revealed significant associations with ECM organization, extracellular structure organization, external encapsulating structure organization, protein localization to the plasma membrane, and regulation of cell size. These pathways are closely linked to cell-matrix interactions and cytoskeletal organization, both of which are essential for proper mitotic spindle positioning and successful cytokinesis. Disruption of ECM-related signaling and membrane-associated protein localization may impair the mechanical and structural coordination required for cell division, ultimately leading to multinucleation and increased cell size. The observed nuclear enlargement further suggests the possibility of polyploidization or altered checkpoint control. Multinucleation and polyploidy are frequently associated with chromosomal instability in aggressive breast cancer (Meléndez-Flórez et al., 2025; Vargas-Rondón et al., 2020), suggesting that LCN2 may contribute to genomic stability in MDA-MB-231 cells. The morphological changes observed upon LCN2 depletion support a role for LCN2 in regulating cellular architecture and mitotic integrity, expanding its functional relevance beyond its established involvement in invasion and metastasis.

The M-FISH analysis confirmed that the generated knockout cell line retains the complex and aneuploid karyotypic architecture characteristic of the parental MDA-MB-231 cell line, without evidence of newly acquired large-scale chromosomal aberrations. This is consistent with a previous report demonstrating that MDA-MB-231 cells exhibit extensive genomic instability and near-triploid karyotypes with numerous structural rearrangements (Varela-Rouco et al., 2025). Importantly, the identification of distinct subclonal populations in the parental cells and the derivation of the knockout line from a defined subclone further support the concept of pronounced intraline heterogeneity in this model system, which has been shown to arise during long-term culture and across different passages (Varela-Rouco et al., 2025). Such heterogeneity, including variations in chromosomal composition and copy number profiles, has been consistently described in independent cytogenetic studies of MDA-MB-231 and other breast cancer cell lines, highlighting ongoing genomic evolution and clonal selection processes (Letessier et al., 2005). Moreover, chromosomal instability in this cell line has been linked to alterations in cytoskeletal organization, further emphasizing its dynamic genomic nature (Dugina et al., 2021). In this context, the absence of additional chromosomal alterations in the LCN2KO cells indicates that the applied genome editing strategy did not exacerbate genomic instability, and that the observed phenotypic differences are likely attributable to the targeted gene disruption rather than to underlying karyotypic changes.

As previously described, light microscopy revealed that LCN2KO cells exhibited a mesenchymal-like morphology. Contrary to expectations from previous studies, the absence of LCN2 in MDA-MB-231 cells did not lead to a shift toward an epithelial morphology (Yang et al., 2009). The study by Yang and colleagues suggested that LCN2 expression promotes a mesenchymal phenotype. Transient siRNA-mediated silencing of LCN2 in MDA-MB-231 cells was reported to reduce vimentin expression, while E-cadherin expression increased. In contrast, stable CRISPR/Cas9-mediated knockout of LCN2 in the present study resulted in decreased *CDH1*/E-cadherin expression, while vimentin expression remained unchanged. A similar reduction in *CDH1* was also observed in LCN2KO PC-3 cells, indicating that this effect is not cell line-specific (Gäberlein et al., 2026). These differences may reflect the distinct biological consequences of transient gene silencing versus permanent genetic ablation, as stable knockout models can undergo long-term cellular adaptation and compensatory changes. Together, these findings suggest that the role of LCN2 in regulating epithelial and mesenchymal markers is context-dependent and may differ according to the duration and mechanism of LCN2 loss. In this study, mesenchymal markers such as *FN1* and *ITGA5* were upregulated. *SNAI2*, a key epithelial-mesenchymal transition (EMT) transcription factor, was downregulated, while other EMT transcription factors and hybrid markers remained largely unchanged at the mRNA level. These findings suggest that the absence of LCN2 did not markedly alter the mesenchymal phenotype or induce a clear EMT/MET shift. It is important to note that EMT was not functionally assessed in this study. Therefore, the differences in EMT-associated marker expression should be interpreted with caution, as these markers are typically dynamic during EMT induction and cellular plasticity processes rather than under steady-state basal culture conditions (Nieto et al., 2016; Yang et al., 2020).

NGS analysis of LCN2KO #15 cells revealed enrichment of pathways associated with ribosome biogenesis, rRNA processing, cytoplasmic translation, and canonical NF-κB signaling. Despite these transcriptional changes, LCN2KO did not enhance proliferative capacity. The CellTiter-Glo® assay demonstrated no significant differences in proliferation between parental and LCN2KO cells. Furthermore, the expression of established proliferation markers *MKI67, TOP2A,* and *CCNA2* remained unchanged. These findings indicate that enrichment of ribosome biogenesis and translational pathways does not necessarily reflect increased cell cycle progression. Instead, the data suggest that LCN2 deficiency is associated with a transcriptional program enriched for ribosome biogenesis and translation-related pathways without promoting proliferation. However, as these observations are based primarily on transcriptomic analyses, and translational regulation may itself be altered following LCN2 loss, further validation at the protein level is required to determine the extent to which transcriptional changes are reflected in protein abundance.

Recent studies have demonstrated that ribosome biogenesis and translational regulation contribute not only to cellular growth but also to adaptive stress responses (Pelletier et al., 2018; Sonenberg and Hinnebusch, 2009). In agreement with the transcriptomic data, which were also validated by RT-qPCR analysis, increased basal expression of IκBα and IκBζ proteins was observed in LCN2KO #15 cells. Furthermore, stimulation with the pro-inflammatory cytokines IL-1β and TNF-α induced IκBζ expression in both parental and #15 cells, confirming activation of the NF-κB pathway. Interestingly, phosphorylation of NF-κB p65 in response to inflammatory stimulation was markedly attenuated in LCN2KO #15 cells despite unchanged total p65 protein levels. However, these analyses were performed after 24 h of cytokine stimulation, whereas phosphorylation of NF-κB p65 is typically a rapid and transient event that occurs within minutes of stimulation. Therefore, the present data do not allow definitive conclusions regarding the early activation kinetics of canonical NF-κB signaling. Instead, they suggest that LCN2 deficiency may alter the sustained response to inflammatory stimulation. Future studies examining earlier time points, e.g., 15–30 min after stimulation, will be required to determine whether LCN2 loss directly influences NF-κB signaling dynamics. Moreover, although RNA-seq demonstrated pronounced transcriptional upregulation of inflammatory mediators such as *CXCL8*, cytokine-induced CXCL8 protein expression was not increased in LCN2KO #15 cells. The discrepancy between transcript and protein expression further suggests that post-transcriptional and translational regulatory mechanisms contribute to the observed phenotype, emphasizing the importance of validating transcriptomic findings at the protein level, particularly in light of the altered translation-associated pathways identified in LCN2KO cells.

Recent studies have highlighted an emerging role for LCN2 in the regulation of iron homeostasis and ferroptosis, particularly in TNBC (Bao et al., 2024). As an iron-binding glycoprotein, LCN2 contributes to cellular iron trafficking and may thereby influence oxidative stress responses and ferroptosis susceptibility (Valashedi et al., 2022). A study by Valashedi et al. showed that, in MDA-MB-231 cells, CRISPR/Cas9-mediated depletion of LCN2 increased sensitivity to erastin-induced ferroptosis and enhanced lipid peroxidation, suggesting that LCN2 functions as a protective factor against ferroptotic cell death. Although the precise molecular mechanisms remain unclear, LCN2 may contribute to ferroptosis resistance through regulation of iron homeostasis and oxidative stress pathways (Valashedi et al., 2022). In the present study, LCN2 deficiency was associated with altered mRNA expression of several genes involved in iron metabolism and ferroptosis, including increased *TFRC*, *FTH1*, *FTL*, and *SLC7A11*, together with reduced *GPX4* and *ACSL4* expression. These findings may suggest that loss of LCN2 is accompanied by transcriptional changes in pathways regulating iron handling and ferroptosis. However, because these observations are based on gene expression analysis alone, they do not establish changes in intracellular iron availability or ferroptotic sensitivity. Moreover, the observed expression profile is not indicative of a straightforward increase or decrease in ferroptosis susceptibility, as some changes (e.g., reduced *ACSL4*) have been associated with decreased ferroptosis sensitivity (Doll et al., 2017), whereas others (e.g., reduced *GPX4*) could have the opposite effect (Yang et al., 2014). Therefore, the functional consequences of these transcriptional alterations remain to be determined. Future studies combining biochemical measurements of iron homeostasis with dedicated ferroptosis assays will be required to clarify whether LCN2 directly modulates ferroptosis in MDA-MB-231 cells. Our findings nevertheless support the emerging concept that LCN2 influences cellular pathways beyond migration and adhesion and may contribute to the regulation of iron metabolism in breast cancer, as proposed in a recent review of LCN2 biology in TNBC (Keller et al., 2025).

Functional assays further supported the involvement of LCN2 in regulating cellular interactions with the extracellular environment. LCN2KO cells displayed altered adhesion behavior depending on the substrate context. Specifically, LCN2KO cells exhibited reduced adhesion under uncoated conditions, whereas adhesion on collagen I was comparable to that of parental cells, and adhesion on Geltrex was increased. This altered adhesion phenotype was accompanied by transcriptional changes in pathways related to the extracellular environment. Transcriptomic analysis revealed significant alterations in ECM organization, extracellular structure organization, protein localization to the plasma membrane, and regulation of cell adhesion. Notably, several integrin genes were upregulated, including *ITGA2* and *ITGA6*, and increased expression of both integrins was confirmed by Western blot analysis. Integrins α2 and α6 mediate interactions with specific ECM components, including collagen- and laminin-containing substrates, respectively (Niland and Eble, 2012). Thus, the increased expression of these integrins may reflect an adaptive response to changes in the extracellular environment following LCN2 loss and may contribute to the substrate-dependent alterations in adhesion observed in LCN2KO cells. However, increased integrin expression does not necessarily translate into increased adhesive strength, as effective cell migration requires dynamic cycles of adhesion formation and disassembly rather than stable adhesion alone (Webb et al., 2002). Consequently, altered integrin expression or signaling could affect the regulation and turnover of cell–matrix adhesions without producing a uniform increase in adhesion across different substrates. Whether ITGA2 or ITGA6 functionally contributes to the observed adhesion phenotype remains to be determined.

Consistent with alterations in cell–matrix interactions, Transwell migration and invasion assays demonstrated significantly reduced migratory and invasive capacity in LCN2KO cells despite unchanged proliferation rates. This finding is in line with a study by Valashedi and colleagues, which reported reduced migration following CRISPR/Cas9-mediated LCN2 knockout using a scratch assay (Valashedi et al., 2022), and with findings by Yang et al., who observed reduced migration following siRNA-mediated LCN2 silencing in MDA-MB-231 cells (Yang et al., 2009). One possible explanation is that altered ECM organization and integrin expression may interfere with the coordinated regulation of adhesion and de-adhesion required for efficient cell movement (Webb et al., 2002; Huttenlocher and Horwitz, 2011). In this context, the increased expression of ITGA2 and ITGA6 may represent a compensatory response that does not restore appropriate adhesion dynamics and may instead be associated with altered cell–matrix signaling. Nevertheless, this interpretation remains speculative, and direct functional evidence is required to establish whether altered integrin-mediated signaling contributes to the migration and invasion phenotype.

To further validate that these phenotypic alterations were directly associated with LCN2 loss, rescue experiments were performed by transient re-expression of human LCN2 in LCN2KO cells, with GFP-transfected cells serving as a transfection control because transfection itself affected cellular behavior. Re-expression of LCN2 partially restored migratory and invasive capacities and improved adhesion under uncoated conditions compared with GFP-transfected LCN2KO cells, supporting a functional role of LCN2 in regulating cell–matrix interactions and migratory and invasive behavior. However, whether these effects involve ITGA2- or ITGA6-dependent mechanisms remains unresolved. Future studies using targeted approaches, such as integrin-blocking antibodies or inhibitors, as well as analyses of focal adhesion signaling and dynamics, will be required to determine whether altered integrin expression and signaling are causally linked to the observed changes in adhesion, migration, and invasion.

In summary, this study demonstrates that LCN2 loss affects cellular behavior, resulting in reduced adhesive, migratory, and invasive capacities, which could be partially restored upon LCN2 re-expression, supporting a direct role for LCN2 in maintaining these processes. Furthermore, LCN2 deficiency altered cellular architecture, including the formation of enlarged cells, indicating a role for LCN2 in regulating cell morphology and cytoskeletal organization. Transcriptomic analysis revealed broad molecular changes upon LCN2 loss, providing insight into the pathways underlying these phenotypic alterations. Together, these findings suggest that LCN2 may contribute to the regulation of cellular adhesion, migration, morphology, and associated gene expression programs in MDA-MB-231 cells.

## 5 Outlook

In this study, we investigated the functional role of LCN2 in the metastatic TNBC cell line MDA-MB-231 using a CRISPR/Cas9-based knockout model. Our results suggest that LCN2 contributes to the regulation of cellular processes associated with metastatic behavior, including mechanisms related to cellular phenotype, stress responses, and interactions with the extracellular environment. These findings highlight a potential role for LCN2 in shaping tumor-associated pathways that may be relevant to TNBC progression.

Further studies should aim to elucidate the molecular mechanisms underlying the observed phenotypic changes upon LCN2 depletion. In particular, the role of integrin-mediated adhesion warrants deeper investigation, including functional validation through targeted inhibition or knockdown approaches. Integrin inhibition experiments could help determine whether the observed alterations in migration, invasion, and adhesion of LCN2-deficient cells are a consequence of dysregulated integrin expression or activity. Furthermore, downstream signaling pathways, including focal adhesion kinase (FAK) activation and focal adhesion dynamics, should be examined to establish whether altered adhesion turnover contributes to the reduced migratory capacity. In addition, future studies should investigate whether LCN2 depletion induces a senescence-like phenotype by assessing established senescence markers, including SA-β-gal activity, p16, p21, γH2AX, and the secretion of SASP-associated cytokines.

To gain further mechanistic insight, it will be important to assess whether adhesion-related transcripts are selectively translated and to distinguish between intracellular and extracellular roles of LCN2 through rescue experiments. Investigating upstream regulatory networks, such as NF-κB signaling, may additionally reveal broader pathways contributing to the observed phenotype. Therefore, short-term stimulation experiments with pro-inflammatory cytokines could be conducted to investigate differences in NF-κB p65 phosphorylation.

Further investigation is required to determine how LCN2-mediated changes in iron metabolism and redox regulation influence ferroptotic susceptibility in TNBC. Such studies may help define the potential of the LCN2–ferroptosis axis as a biomarker for tumor biology and as a target for future therapeutic strategies. Moreover, it will be necessary to determine whether the phenotypes associated with LCN2 deficiency observed in the MDA-MB-231 model are conserved across distinct TNBC cellular backgrounds. In particular, validation of LCN2 loss and rescue phenotypes in an independent TNBC cell line with a different genetic background would help establish the broader relevance and generalizability of the findings reported here. Such studies may also help determine whether the cellular response to LCN2 deficiency is influenced by differences in basal LCN2 expression or other cell-line-specific characteristics.

Lastly, extending these analyses to more physiologically relevant systems, such as three-dimensional spheroid models cultured with ECM components, will allow investigations of cell-matrix interactions, adhesion, and invasive behavior in an environment that better mimics the tumor microenvironment. Ultimately, *in vivo* metastasis models will be essential to validate the translational relevance of LCN2-dependent mechanisms in TNBC progression.

## Data Availability

The datasets generated and/or analyzed during the current study are available in the Gene Expression Omnibus (GEO) repository, https://www.ncbi.nlm.nih.gov/geo/query/acc.cgi?acc=GSE328115, accession number GSE328115.
