# Supplementary material for: Comprehensive characterization of the cellular and molecular consequences of LCN2 loss in MDA-MB-231 triple-negative breast cancer cells
Source: Front Cell Dev Biol. 2026 Oct 21;14:1909534. doi: 10.3389/fcell.2026.1909534 (PMC13749177; doi:10.3389/fcell.2026.1909534)
Supplement: Supplementary file 1 [file Supplementaryfile1.docx]

**Supplementary Table 1: Primers used in ihis study**

| **Gene** | **Accession No.** | **Forward primer (5'->3')** | **Location** | **Reverse primer (5'->3')** | **Location** | **Amplicon size [bp]** | **Reference** |
| --- | --- | --- | --- | --- | --- | --- | --- |
| *ACTA2* | NM_001613.4 | CTATGCCTCTGGACGCACAACT | 498-519 | CAGATCCAGACGCATGATGGCA | 612-591 | 114 | #HP205437, Origene, Rockville, MD, USA |
| *CASP1* | NM_033292.4 | CCTTAATATGCAAGACTCTCAAGGA | 325-349 | TAGCTGGGTTGTCCTGCACT | 398-379 | 74 | NCBI primer design tool |
| *CCNA2* | NM_001237.5 | CTCTACACAGTCACGGGACAAAG | 1345-1367 | CTGTGGTGCTTTGAGGTAGGTC | 1464-1443 | 119 | #HP205163, Origene |
| *CDH1* | NM_004360.4 | CCCGGGACAACGTTTATTAC | 2367-2386 | GCTGGCTCAAGTCAAAGTCC | 2438-2419 | 71 | NCBI primer design tool |
| *CXCL1* | NM_001511.4 | AGTGGCACTGCTGCTCCT | 129-146 | TGGATGTTCTTGGGGTGAAT | 251-232 | 123 | [1] |
| *FN1* | NM_212482.4 | CCACCCCCATAAGGCATAGG | 6736-6755 | GTAGGGGTCAAAGCACGAGTCATC | 7169-7146 | 433 | [2] |
| *GAPDH* | NM_001289746.2 | AGCCACATCGCTCAGACAC | 57-75 | GCCCAATACGACCAAATCC | 122-104 | 66 | NCBI primer design tool |
| *IL1B* | NM_000576.3 | TACCTGTCCTGCGTGTTGAA | 637-656 | TCTTTGGGTAATTTTTGGGATCT | 712-690 | 76 | NCBI primer design tool |
| *ITGA2* | NM_002203.4 | TTGCGTGTGGACATCAGTCTGG | 2383-2404 | GCTGGTATTTGTCGGACATCTAG | 2540-2518 | 157 | #HP205938, Origene |
| *ITGA6* | NM_001316306.2 | CGAAACCAAGGTTCTGAGCCCA | 1556-1577 | CTTGGATCTCCACTGAGGCAGT | 1706-1685 | 150 | #HP230173, Origene |
| *LCN2* | NM_005564.5 | CTCCACCTCAGACCTGATCC | 139-158 | ACATACCACTTCCCCTGGAAT | 231-211 | 93 | NCBI primer design tool |
| *MKI67* | NM_002417.5 | GAAAGAGTGGCAACCTGCCTTC | 2270-2291 | GCACCAAGTTTTACTACATCTGCC | 2420-2397 | 150 | #HP206104, Origene |
| *NFKBIA* | NM_020529.2 | GTCAAGGAGCTGCAGGAGAT | 241-260 | GATGGCCAAGTGCAGGAA | 351-334 | 111 | NCBI primer design tool |
| *TOP2A* | NM_001067.4 | GTGGCAAGGATTCTGCTAGTCC | 2492-2513 | ACCATTCAGGCTCAACACGCTG | 2626-2605 | 134 | #HP203788, Origene |
| *VIM* | NM_003380.3 | TACAGGAAGCTGCTGGAAGG | 1629-1648 | ACCAGAGGGAGTGAATCCAG | 1732-1713 | 103 | NCBI primer design tool |

References

1.	Sanzari, J.K.; Nuth, M.; Kennedy, A.R. Induction of cytokine gene expression in human thyroid epithelial cells irradiated with HZE particles (iron ions). *Radiat. Res.* **2009**, *172*, 437–443, doi:10.1667/RR1363.1.

2.	Tancred, T.M.; Belch, A.R.; Reiman, T.; Pilarski, L.M.; Kirshner, J. Altered expression of fibronectin and collagens I and IV in multiple myeloma and monoclonal gammopathy of undetermined significance. *J. Histochem. Cytochem.* **2009**, *57*, 239–247, doi:10.1369/jhc.2008.952200.

| **Supplementary Table 2: Primary and secondary antibodies used for Western blot analysis** | | | | | |
| --- | --- | --- | --- | --- | --- |
| **Antibody** | **Catalog No.** | **RRID^1^** | **Dilution** | **Host, Clonality** | **Company** |
| CXCL8 (C-11) | sc-376750 | AB_2891256 | 1:1000 | m, mAb | Santa Cruz Biotech., Dallas, TX, USA |
| E-Cadherin (24E10) | 3195 | AB_2291471 | 1:1000 | r, mAb | Cell Signaling Technology, Danvers, MA, USA |
| Fibronectin | AB1954 | AB_2105708 | 1:3000 | r, pAb | Sigma-Aldrich, Taufkirchen, Germany |
| GAPDH (6C5) | sc-32233 | AB_627679 | 1:1000 | m, mAb | Santa Cruz Biotech. |
| GFP (B-2) | sc-9996 | AB_627695 | 1:1000 | m, mAb | Santa Cruz Biotech |
| IκBα | sc-371 | AB_2235952 | 1:1000 | r, pAb | Santa Cruz Biotech |
| IκBζ | 9244 | AB_2151602 | 1:1000 | r, pAb | Cell Signaling |
| ITGA2 | 88228 | AB_3753417 | 1:1000 | r, pAb | Cell Signaling |
| ITGA6 | 3750 | AB_2249263 | 1:1000 | r, pAb | Cell Signaling |
| Keratin 19 (D7F7W) | 13092 | AB_2722626 | 1:1000 | r, mAb | Cell Signaling |
| Lipocalin-2 | AF1757 | AB_354974 | 1:1000 | g, pAb | R&D Systems, Minneapolis, MN, USA |
| NF-κB p65 (F-6) | sc-8008 | AB_628017 | 1:1000 | m, mAb | Santa Cruz Biotech |
| NF-κB p65 phsophorylated Ser536 (93H1) | 3033L | AB_331284 | 1:1000 | r, mAb | Cell Signaling |
| Turbo-green fluorescent protein (OTI2H8) | TA150041 | AB_2622256 | 1:1000 | m, mAb | Origene, Rockville, MD, USA |
| Vimentin (EPR3776) | ab92547 | AB_10562134 | 1:3000 | r, mAb | Abcam, Cambridge, Great Britain |
| α smooth muscle actin antibody [EPR5368] | ab124964 | AB_11129103 | 1:1000 | r, mAb | Abcam |
| β-actin | A5441 | AB_476744 | 1:10,000 | m, mAb | Sigma-Aldrich |
| **Secondary antibodies** | | | | | |
| mouse anti-goat IgG (H+L), HRP | 31400 | AB_228370 | 1:5000 | m, pAb | Thermo Fisher Scientific, Waltham, MA, USA |
| goat anti-mouse IgG  (H+L), HRP | 31430 | AB_228307 | 1:5000 | g, pAb | Thermo Fisher Scientific |
| goat anti-rabbit IgG  (H+L), HRP | 31460 | AB_228341 | 1:5000 | g, pAb | Thermo Fisher Scientific |
| **Abbreviations used are**: pAb, polyclonal antibody; mAb, monoclonal antibody; g, goat; m, mouse; r, rabbit. ^1^Data were obtained from the Research Resource Identifier (RRID) portal, which is available at https://www.rrids.org/. | | | | | |

**Supplementary Table 3: Short Tandem Repeat Profiling in MDA-MB-231 and MDA-MB-468**

| **Marker** | **Sample**  **MDA-MB-231** | **Reference**  **MDA-MB-231*** | **Sample**  **MDA-MB-468** | **Reference**  **MDA-MB-468**** |
| --- | --- | --- | --- | --- |
| AMEL | X | X | X | X |
| CSF1PO | 12,13 | 12,13 | 12 | 12 |
| D13S317 | 13 | 13 | 12 | 12 |
| D16S539 | 12 | 12 | 9 | 9 |
| D18S51 | 11 | 11,16 | 17 | 17 |
| D21S11 | 30,33.2 | 33.2 | 27,28 | 27,28 |
| D3S1358 | 16 | 16 | 15 | 15 |
| D5S818 | 12 | 12 | 12 | 12 |
| D7S820 | 8, 9 | 8, 9 | 8 | 8 |
| D8S1179 | 13 | 13 | 13 | 13 |
| FGA | 22,23 | 22,23 | 23 | 23 |
| Penta_D | 11,14 | 11,14 | 8,10 | 8,10 |
| Penta_E | 11 | 11 | 5 | 5 |
| TH01 | 7,9.3 | 7,9.3 | 7 | 7 |
| TPOX | 8,9 | 8,9 | 8,9 | 8,9 |
| vWA | 15,18 | 15,18 | 18 | 18 |
| **Identity Match** | **> 80%** | | **100%** | |

* Data was taken from Yu M, Selvaraj SK, Liang-Chu MM, Aghajani S, Busse M, Yuan J, Lee G, Peale F, Klijn C, Bourgon R, Kaminker JS, Neve RM. Nature 2015;520(7547):307-11. doi: 10.1038/nature14397. ** The data was sourced from the Cellosaurus database, which can be accessed at: https://www.cellosaurus.org/CVCL_0419. The chromatograms for this analysis can be found in Supplementary Figure 3 for MDA-MB-231 and Supplementary Figure 4 for MDA-MB-468. Any marker information that differs from other sources is highlighted in red. For more information, please refer to the text

| **Supplementary Table 5 Differentially expressed genes across parental and LCN2KO cells** | | | | | |  |
| --- | --- | --- | --- | --- | --- | --- |
| **Gene ID** | **Gene** | **TPM Parental** | **TPM Knockout** | **Log2 FoldChange** | **padjust.** | **Significance** |
| **Mesenchymal marker** | | | | | | |
| ENSG00000107796.15 | *ACTA2* | 24.70009976  28.11146525  33.26427492 | 26.42781328  12.46577443  19.03920931 | -0.562566376 | 0.447895715 | no |
| ENSG00000170558.11 | *CDH2* | 7.600030697  1.813642919  1.073041127 | 5.663102845  1.038814536  4.230935401 | 0.058156586 | 0.986144483 | no |
| ENSG00000115414.22 | *FN1* | 788.5031848  658.3523797  983.978713 | 1193.026999  980.640922  1115.909212 | 0.438925007 | 0.001498131 | yes |
| ENSG00000161638.12 | *ITGA5* | 266.9510782  249.3759014  268.2602816 | 334.1230678  325.1489498  319.4356228 | 0.320149633 | 0.051381783 | no |
| ENSG00000026025.17 | *VIM* | 14773.50967  13989.53466  16079.52128 | 14939.2653  15950.9972  14831.54405 | 0.028174931 | 0.812530752 | no |
| **Epithelial marker** | | | | | |  |
| ENSG00000039068.21 | *CDH1* | 71.25028778  62.57068072  65.45550872 | 16.04545806  10.38814536  15.86600775 | -2.232920493 | 3,03247∙10^-8^ | yes |
| ENSG00000163347.6 | *CLDN1* | 87.40035301  43.52743006  79.40504336 | 153.8476273  116.347228  144.9095375 | 0.986453622 | 0.000734957 | yes |
| ENSG00000119888.12 | *EPCAM* | 76.00030697  67.10478801  106.2310715 | 67.01338366  93.49330825  67.69496642 | -0.123770255 | 0.828117137 | no |
| ENSG00000170421.14 | *KRT8* | 2597.310491  2626.154947  2889.699754 | 2171.799941  2195.015115  2134.50691 | -0.318831625 | 3.05053∙10^-6^ | yes |
| ENSG00000111057.12 | *KRT18* | 2830.061431  3025.156389  3363.983932 | 2520.080766  2674.94743  2747.992543 | -0.214653752 | 0.010739622 | yes |
| ENSG00000197822.13 | *OCLN* | 124.4505027  119.7004327  110.523236 | 136.8583187  149.5892932  129.0435297 | 0.226979081 | 0.476037408 | no |
| **Epithelial-mesenchymal transition** | | | | | | |
| ENSG00000124216.4 | *SNAI1* | 3.800015348  9.068214596  6.438246759 | 5.663102845  3.116443608  6.346403102 | -0.353502781 | 0.857017853 | no |
| ENSG00000019549.13 | *SNAI2* | 64.60026092  48.06153736  93.35457801 | 24.54011233  21.81510526  25.38561241 | -1.516006759 | 0.000156138 | yes |
| ENSG00000148516.24 | *ZEB1* | 298.3012048  276.5805452  315.4740912 | 261.4465813  283.5963683  270.7798657 | -0.12517521 | 0.603158968 | no |
| ENSG00000169554.24 | *ZEB2* | 36.10014581  48.06153736  46.14076844 | 55.68717797  49.86309773  52.88669251 | 0.283266796 | 0.615051818 | no |
| **Plasticity** | | | | | | |
| ENSG00000167601.13 | *AXL* | 3885.515694  4164.124143  3844.706356 | 3227.024771  3386.535388  3441.865949 | -0.242901164 | 0.000139474 | yes |
| ENSG00000026508.22 | *CD44* | 2712.260955  2626.154947  2720.159256 | 3193.046154  3367.836726  3195.413962 | 0.275921352 | 5.75329∙10^-6^ | yes |
| ENSG00000106799.16 | *TGFBR1* | 377.1515233  381.7718345  371.2722298 | 414.3503581  369.8179748  433.6708786 | 0.107519122 | 0.612108634 | no |
| ENSG00000163513.21 | *TGFBR2* | 361.9514619  381.7718345  444.2390264 | 383.2032925  393.7107092  410.4007339 | 0.000808499 | 0.998783144 | no |
| **Iron metabolism** | | | | | | |
| ENSG00000068366.22 | *ACSL4* | 840.7533958  848.7848862  798.3425981 | 369.9893859  341.7699824  322.6088243 | -1.265219793 | 3.67072∙10^-36^ | yes |
| ENSG00000167996.17 | *FTH1* | 12080.24879  10851.93241  13047.10706 | 24261.67644  21730.96128  22491.65259 | 0.928868824 | 4,49254∙10^-48^ | yes |
| ENSG00000087086.17 | *FTL* | 13691.4553  12752.63019  13991.38325 | 17144.10001  17424.03621  16777.77433 | 0.344797082 | 6.99407∙10^-12^ | yes |
| ENSG00000167468.20 | *GPX4* | 1678.65678  1636.812735  1961.519179 | 1398.786403  1245.538629  1148.698961 | -0.474631169 | 7.66395∙10^-7^ | yes |
| ENSG00000266412.7 | *NCOA4* | 844.5534112  867.8281369  774.7356933 | 766.406585  842.4785887  745.7023645 | -0.080227608 | 0.627060187 | no |
| ENSG00000151012.13 | *SLC7A11* | 251.7510168  184.0847563  245.726418 | 364.326283  310.6055463  486.5575711 | 0.770260083 | 4.91453∙10^-5^ | yes |
| ENSG00000138449.12 | *SLC40A1* | 3.800015348  8.161393137  11.80345239 | 8.494654267  4.155258144  2.115467701 | -0.665746566 | 0.699178083 | no |
| ENSG00000072274.15 | *TFRC* | 1634.0066  1636.812735  1355.250943 | 2024.559267  2210.597333  2071.042879 | 0.445538727 | 1.59037∙10^-7^ | yes |
| **Oxidative stress** | | | | | | |
| ENSG00000023909.11 | *GCLM* | 385.7015579  410.7901212  310.1088856 | 532.3316674  606.6676891  529.924659 | 0.589507238 | 1.24575∙10^-5^ | yes |
| ENSG00000100292.18 | *HMOX1* | 60.80024557  64.38432363  53.65205633 | 71.73263603  76.87227567  49.71349096 | 0.148601948 | 0.799655676 | no |
| ENSG00000079999.15 | *KEAP1* | 398.0516077  408.9764783  324.0584202 | 424.7327134  573.4256239  514.0586512 | 0.415516436 | 0.009321765 | yes |
| ENSG00000116044.18 | *NFE2L2* | 494.0019953  501.4722672  441.019903 | 575.7487892  502.7862355  503.4813127 | 0.138870216 | 0.430709852 | no |
| ENSG00000181019.13 | *NQO1* | 346.7514005  409.8832998  351.9574895 | 560.6471816  631.5992379  601.8505608 | 0.692360026 | 5,21039∙10^-9^ | yes |
| ENSG00000142168.16 | *SOD1* | 2014.008135  2093.85075  1966.884385 | 1403.505655  1590.425055  1477.654189 | -0.442941563 | 1.44577∙10^-9^ | yes |
| ENSG00000291237.2 | *SOD2* | 1814.507329  1389.250476  1657.84854 | 3508.292212  2559.639017  2979.636256 | 0.896343959 | 3.18074∙10^-6^ | yes |
| **Inflammation** | | | | | | |
| ENSG00000169429.13 | *CXCL8* | 40.850165  12.69550044  31.11819267 | 1227.005616  867.4101376  1040.810109 | 5.214918112 | 2.37077∙10^-31^ | yes |
| ENSG00000125538.12 | *IL1B* | 31.35012662  13.60232189  33.26427492 | 558.7594807  333.4594661  424.151274 | 4.080501964 | 3.89092∙10^-21^ | yes |
| ENSG00000136244.13 | *IL6* | 70.30028395  72.54571677  74.03983773 | 206.7032538  195.2971328  192.5075608 | 1.45593133 | 2.81829∙10^-13^ | yes |
| ENSG00000100906.13 | *NFKBIA* | 552.9022332  428.9265504  550.4700979 | 1536.588572  1384.739777  1370.82307 | 1.488557383 | 7.94447∙10^-51^ | yes |
| ENSG00000144802.11 | *NFKBIZ* | 104.5004221  61.66385926  89.0624135 | 231.2433662  246.199045  252.7983902 | 1.519539989 | 8.29627∙10^-14^ | yes |
| ENSG00000173039.21 | *RELA* | 699.2028241  652.0046295  698.5497734 | 874.9493895  779.110902  818.6860001 | 0.271931084 | 0.012182884 | yes |
| ENSG00000168610.18 | *STAT3* | 785.6531733  882.3372802  801.5617215 | 958.0082312  912.0791627  942.4408606 | 0.187066893 | 0.094635774 | no |
| ENSG00000148346.13 | *LCN2* | 64.60026092  36.27285839  52.5790152 | 507.7915551  335.5370951  488.6730388 | 3.122422955 | 1.1635E-50 | yes |

Annotation: TPM values for parental MDA-MB-231 and knockout cells LCN2KO #15, each derived from three independent experiments (n = 3 per cell line). Differential gene expression was calculated using parental cells as the reference (baseline) group and LCN2KO cells as the comparison group. Therefore, positive log2 fold-change (log2FC) values indicate genes upregulated in LCN2KO cells relative to parental cells, whereas negative log2FC fold-change values indicate genes downregulated in LCN2KO cells.

**Supplementary Figure 1:** Chromatograms of short tandem repeat (STR) markers used for MDA-MB-231 cell line authentication. Peaks in the chromatograms indicate the fragment sizes of alleles detected in the sample. These fragment sizes were used to determine the allele calls that are summarized in Supplementary Table 4.

**Supplementary Figure 2:** Chromatograms of short tandem repeat (STR) markers used for MDA-MB-468 cell line authentication. Peaks in the chromatograms indicate the fragment sizes of alleles detected in the sample. These fragment sizes were used to determine the allele calls that are summarized in Supplementary Table 4.

**Supplementary Figure 3: Stimulation of MDA-MB-231 cells with pro-inflammatory cytokines**. Representative Western blot of protein extracts from MDA-MB-231 cells treated with or without 2.5 ng/ml IL-1β, 10 ng/mL TNF-α or 2.5 ng/mL TGF-β1. Equal protein loading was verified by β-Actin and Ponceau S stain. All experiments were performed in biological triplicates (n=3). Original uncropped blots are presented in **WB7**.

**Supplementary Figure 4: Verification of LCN2 knockout in MDA-MB-231 clones at the protein level**. Western blot analysis of protein extracts from the parental MDA-MB-231 cells and all LCN2 knockout clones generated using three different approaches (LCN2KO 1-3). Equal protein loading was confirmed by β-Actin (n=1). Original uncropped blots are presented in **WB8**.

**Supplementary Figure 5: Verification of LCN2 knockout in MDA-MB-231 clones at the mRNA level.** *LCN2* mRNA expression in the parental MDA-MB-231 cells and all LCN2 knockout clones generated using three approaches (LCN2KO 1-3) are shown. Data are presented as the mean ± standard deviation. The experiment was performed with technical replicates (n=3).

**Supplementary Figure 6:** Brightfield and fluorescence microscopy pictures of (A) parental MDA-MB-231 and (B) LCN2KO #15 cells. Scale bars show 250 µm at 100× and 200× magnification.

**Supplementary Figure 7: Quantification of enlarged cell formation in LCN2KO #15 cells.** Three independent fields of view from brightfield microscopy images of parental MDA-MB-231 cells and LCN2KO #15 were analyzed. Enlarged cells were defined as cells exhibiting an area at least two-fold greater than the average area of parental cells. (A) Percentages of enlarged cells in parental MDA-MB-231 cells and LCN2KO #15 cells. Normality was assessed using the Shapiro–Wilk test, and statistical significance was determined by *t*-test. Statistical significance is indicated by asterisks, ** *p*<0.01. (B) Representative brightfield microscopy images of parental MDA-MB-231 and LCN2KO #15 cells used for analysis are shown. (C) Brightfield microscopy images of parental MDA-MB-231 and LCN2KO #15 cells acquired at 40 × magnification. (D) Brightfield images of additional LCN2KO clones (LCN2KO3 #2, LCN2KO1 #4, and LCN2KO3 #10), which were not included in the quantitative morphological analysis, are shown as representative examples. The scale bar represents 250 µm or 500 µm.

Supplementary Figure 8: Analysis of inflammatory markers and NF-κΒ pathway in LCN2KO cells. (A) Relative mRNA expression of inflammation-associated markers *NFKBIA, CASP1, CXCL1,* and *IL1B* in parental MDA-MB-231 cells and LCN2KO clones (#7, #12, and #15). All experiments were conducted in three independent replicates (n=3). Data is presented as the mean ± SD. (B) Representative Western blot of protein extracts from parental MDA-MB-231 cells and LCN2KO #15 treated with IL-1β, TNF-α, TGF-β1, and IL-6. Original uncropped blots are presented in WB9. Normality was assessed using the Shapiro–Wilk test, and statistical significance was determined by ordinary one-way ANOVA with Dunnett’s multiple comparisons test or the Kruskal-Wallis test with Dunn’s multiple comparisons test. Statistical significance is denoted with asterisks: * *p*<0.05, ** *p*<0.01, *** *p*<0.001.

**Supplementary Figure 9: Re-expression of LCN2 in LCN2KO #15.** (A) Representative Western Blot analysis of protein extracts from parental MDA-MB-231 and LCN2KO #15 transfected with pCMV-Sport6-hLCN2 (*LCN2*) or pEGFP-N1 (*GFP*). Equal protein loading was verified by β-actin (n=1). Original uncropped blots are presented in **WB10**. As the strong LCN2 signal in LCN2-transfected MDA-MB-231 cells limited the visualization of the weaker basal endogenous LCN2 signal in the original Western blot, lysate from untransfected parental MDA-MB-231 cells was re-probed under detection conditions optimized for basal LCN2 expression. (B+C+D) LCN2KO #15 cells were transiently transfected with either pCMV-SPORT6-hLCN2 (*LCN2*) or pEGFP-N1 (*GFP*). (B) Migration and (C) invasion assays were performed and migrated/invaded cells were stained with 0.2% (w/v) crystal violet solution. Migrated/invaded cells were quantified using ImageJ (1.54p). Data is shown as mean ± SD (n=3). (D) Cell adhesion was assessed by crystal violet staining, and the absorbance of solubilized dye was measured at 590 nm. Data is shown as the mean ± SD (n=3). Normality was assessed using the Shapiro–Wilk test, and statistical significance was determined by *t*-test. Statistical significance is indicated by asterisks, * p<0.05, *** p<0.001. Representative images of three independent experiments showing (E) migration and (F) invasion of LCN2KO #15 cells transfected with pEGFP-N1 (*GFP)* or pCMV-Sport6-hLCN2 (*LCN2*) through hanging inserts with an 8.0 µm pore size. Migrated/invaded cells were stained with 0.2% (w/v) crystal violet solution.

β-Actin

Chemi-image

GAPDH

**Supplementary Figure 10: Proliferation analysis of parental MDA-MB-231 cells and LCN2KO #15 cells.** (A) Cell proliferation was evaluated indirectly by measuring intracellular ATP levels with the CellTiter-Glo^®^ 3D Cell Viability Assay at 24 and 48 h after seeding. (B) mRNA expression of *MKI67, TOP2A,* and *CCNA2* in parental MDA-MB-231 cells and knockout clones #7, #12 and #15. All experiments were conducted in biological triplicates (n=3). Data is presented as the mean ± standard deviation. Normality was assessed using the Shapiro–Wilk test, and statistical significance was determined by ordinary one-way ANOVA with Dunnet’s multiple comparison test. Statistical significance is denoted with asterisks: * *p*<0.05.

**Uncropped Western Blot (WB) Images**

**WB1** Full, uncropped immunoblot corresponding to Figure 1B (LCN2, turbo-GFP, β-Actin). Molecular weight markers and membrane overlay are shown.

**WB2** Full, uncropped immunoblot corresponding to Figure 1D (LCN2, Vimentin, β-Actin). Molecular weight markers and membrane overlay are shown. Although Vimentin was not presented as a separate panel in Figure 1D, it was detected on the same membrane alongside β-actin and is shown separately here for completeness.

**WB3** Full, uncropped immunoblot corresponding to Figure 6A Blot 1 (FN1, Keratin19, β-Actin, GAPDH). Molecular weight markers and membrane overlay are shown.

**WB4** Full, uncropped immunoblot corresponding to Figure 6A Blot 2 (α-SMA, GAPDH). Molecular weight markers and membrane overlay are shown.

**WB5** Full, uncropped immunoblot corresponding to Figure 6A Blot 3 (E-Cadherin, Vimentin, LCN2, GAPDH). Molecular weight markers and membrane overlay are shown.

**WB6** Full, uncropped immunoblot corresponding to Figure 6F. Molecular weight markers and membrane overlay are shown.

**WB7** Full, uncropped immunoblot corresponding to Supplementary Figure 3 (LCN2, E-Cadherin, Vimentin, β-Actin). Molecular weight markers and membrane overlay are shown.

**WB8** Full, uncropped immunoblot corresponding to Supplementary Figure 4 (LCN2, turbo-GFP, β-Actin). Molecular weight markers and membrane overlay are shown.

**WB9** Full, uncropped immunoblot corresponding to Supplementary Figure 8. Molecular weight markers and membrane overlay are shown.

**WB10** Full, uncropped immunoblot corresponding to Supplementary Figure 9A. Molecular weight markers and membrane overlay are shown. Untransfected parental MDA-MB-231 cells were reprobed to detect LCN2 expression. The corresponding LCN2 and β-actin bands are indicated by *. Additional bands visible in the blot are from a separate experiment and are unrelated to the present analysis.
